# Male reproductive cycle in a population of the endemic butterfly lizard, *Leiolepis ocellata* Peters, 1971 (Squamata: Agamidae) from northern Thailand

**DOI:** 10.1186/s40850-022-00145-6

**Published:** 2022-08-05

**Authors:** Akkanee Pewhom, Thidaporn Supapakorn, Nopparat Srakaew

**Affiliations:** 1 Department of Biology, Faculty of Science, Thaksin University, Phatthalung, 93210 Thailand; 2 Department of Statistics, Faculty of Science, Kasetsart University, Bangkok, 10900 Thailand; 3 Department of Zoology, Faculty of Science, Kasetsart University, Bangkok, 10900 Thailand

**Keywords:** Butterfly lizard, Reproductive cycle, Histomorphology, Histochemistry, Testis, Male genital duct, Sex hormone, Thailand

## Abstract

**Background:**

Fundamental knowledge on the seasonal reproductive microanatomy and endocrinology of reptiles has been collected from several studies of various species. The present study was to determine annual changes in hormonal profiles, and detailed histomorphometric and histochemical characteristics of the entire male reproductive system of the tropical agamid lizard, *Leiolepis ocellata*.

**Results:**

Male *L. ocellata* individuals (*n* = 75) collected from the territory of two provinces (Lampang and Tak) in northern Thailand exhibited annual variation in sex hormonal, histomorphometric, and histochemical characteristics of the male reproductive system. The reproductive cycle was subdivided into eight reproductive periods (early first active, first active, resting, second recrudescent, second active, regressive, quiescent, and first recrudescent), thus displaying a bimodal pattern with two actively reproductive periods. Circulating sex hormones (testosterone, estradiol, and progesterone) peaked in the first active (February) and the second active (June–July) periods. Likewise, gonadosomatic index (GSI) and histomorphometric variables of the testes and of the genital ducts (rete testis, ductuli efferentes, ductus epididymis, and ductus deferens) revealed their highest values in the first active period. Marked increase in protein and carbohydrate production was detectable in the ductuli efferentes during the active periods.

**Conclusions:**

The male reproductive cycle of *L. ocellata* showed a biannual pattern of the hormonal profile, and detailed histomorphometric and histochemical characteristics of the entire reproductive system. Hence, the present study provides improved basic knowledge on the reptilian reproductive biology with comparative viewpoints to other reptiles.

**Supplementary Information:**

The online version contains supplementary material available at 10.1186/s40850-022-00145-6.

## Background

Male lizards generally display three patterns of the reproductive cycles, viz. continuous (constant), associated (synchronous), and dissociated (asynchronous) patterns [1]. The continuous reproductive cycle is found in tropical lizards that have spermatogenesis and mating throughout the year, concurrent with complete vitellogenesis in females [1–4]. In the associated type, maximal gametogenesis of males and females is seasonally synchronous and coincides with copulatory behavior [1, 5]. This pattern is common in species inhabiting temperate zones [6, 7], but it can also be found in tropical and subtropical lizards [8, 9]. The dissociated reproductive cycle is typical of lizards in temperate regions where males and females produce mature gametes asynchronously [10–13]. Mating periods are short and, thus, spermatozoa are retained for subsequent fertilization in the sperm storage tubules that are modified from male or female reproductive tracts [11, 13–15].

Endocrine regulations are key determinants influencing the reproductive cycles of male lizards [16]. Plasma testosterone (T) levels are correlated with spermatogenic activities and exhibit seasonal cyclicity in that T concentrations are low during early stages of spermatogenesis (quiescent and recrudescent periods), gradually elevate and peak during spermiogenesis (active period), coincident with full development of male genital ducts and of renal sexual segments, and with breeding [17, 18]. Circulating estradiol (E_2_) and progesterone (P) also display a seasonal cycle in male lizards, with their increased levels during mating period and their declined levels during non-mating, quiescent, and recrudescent periods [17, 19]. It is suggested that E_2_ is implicated in sexual behaviors and testicular growth, while P may stimulate reproductive behaviors in male lizards [17, 20, 21].

Microanatomical and histochemical profiles of the male reproductive system in reptiles reveal their seasonal variation [13, 22–32]. In addition, information on correlation among detailed reproductive microanatomy and endocrinology has been gathered from several studies of various reptilian species [22, 30, 33, 34]. Nonetheless, seasonal patterns of detailed histomorphological and histochemical characteristics of the entire male reproductive system from a single reptilian species are still not available.

The butterfly lizard, *Leiolepis ocellata*, is endemic to northern Thailand [35, 36]. This lizard species is diurnal and mostly found in the deciduous forests, likely in areas with an average temperature of 26.49 °C, an average relative humidity of 72.68%, and an average daily rainfall of 6.80 mm [35]. They are omnivorous and feed on insects and young plant shoots [35]. Information on the reproductive biology of *L. ocellata* is scarce, with the only recent microscopic descriptions of the male reproductive system during the active period [37]. However, it remained questionable whether the reproductive structures and sex hormones of *L. ocellata* undergo seasonal cyclicity. We hypothesized that, like other reptiles, the reproductive system and sex hormonal profiles of *L. ocellata* displayed seasonal variation.

The present study aimed to determine the annual cycle of the reproductive histomorphology, histochemistry, and endocrinology of the tropical agamid lizard, *L. ocellata*.

## Results

**Fig. 1:**
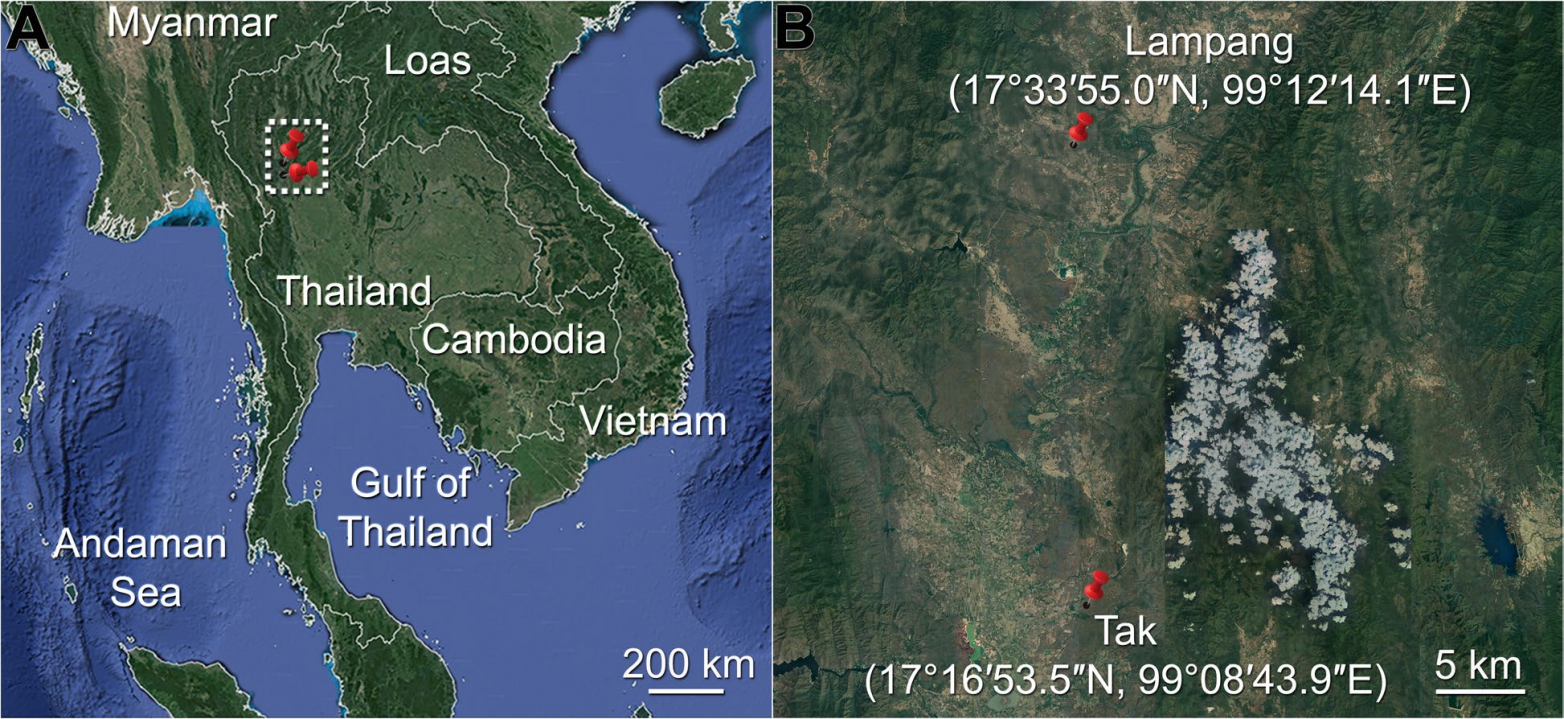
Sampling locations. **A** Map of Thailand showing two sampling sites (dashed rectangle) in northern Thailand. **B** Enlarged map from the rectangle in (**A)** revealing the two sampling sites in Tak and Lampang provinces with approximately 32 km apart. Images were created by using Google Map©2022

**Fig. 2:**
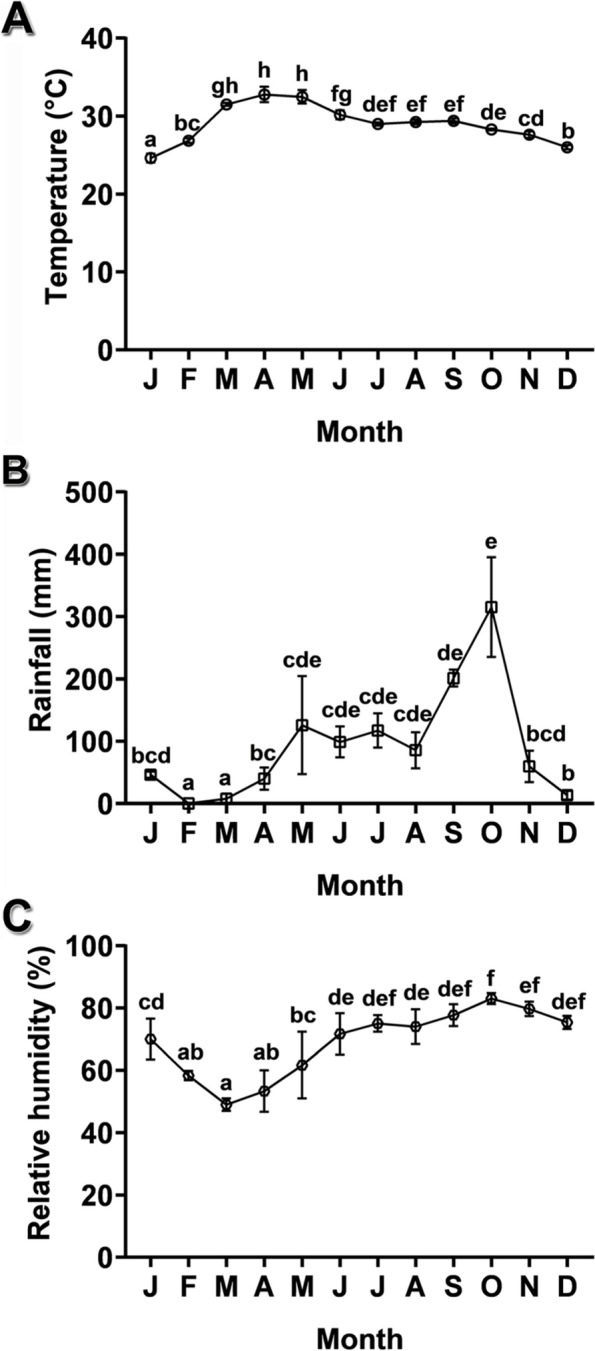
Physical parameters over the three-year period (2015–2017) in the study areas. **A** Monthly temperature. **B** Monthly rainfall. **C** Monthly relative humidity. Data are presented as mean ± SE (*n* = 3). Different superscript letters of the values represent significant differences (*P* < 0.05)

Two sampling localities in Lampang and Tak provinces are approximately 32 km apart (Fig. 1). Mean annual air temperature, rainfall, and humidity were 28.99 °C, 92.6 mm, and 69%, respectively (Fig. 2). In general, the climate of the study areas consists of three seasons. The winter, which runs from mid-October to mid-February, is cold and dry, and has minimal precipitation, with the coldest in December and January. The temperature rises in the summer (mid-February to mid-May) with low precipitation, and the hottest in April. The monsoon season runs from May to October, with heavy rain and cooler temperatures during the day than in the summer.

The lowest and the highest mean temperature was 24.60 °C (January) and 32.78 °C (April), respectively (Fig. 2A). The lowest and the highest mean rainfall was 0 mm (February) and 315.13 mm (October), respectively (Fig. 2B). The lowest and the highest mean humidity was 49% (March) and 83% (October), respectively (Fig. 2C).

### Reproductive cycle of male *Leiolepis ocellata*

**Fig. 3:**
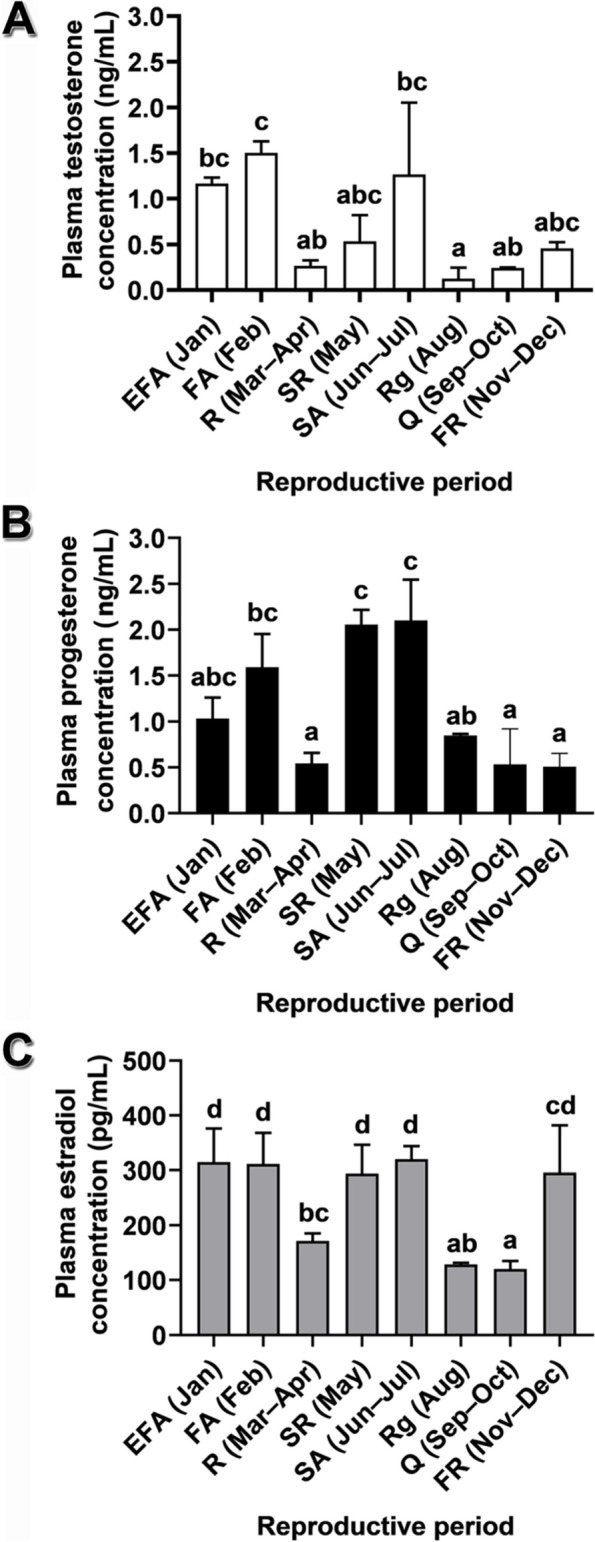
Annual profile of plasma sex steroids at different reproductive periods of *Leiolepis ocellata*. **A** Testosterone. **B** Progesterone. **C** Estradiol. Each value of hormone concentrations was obtained from an average of plasma hormones of four randomly selected individuals in the same reproductive period, with the exception in January (early first active period) having three individuals, and presented as mean ± SE. Different letters denote significant differences of sex steroid levels among reproductive periods (*P* < 0.05). Abbreviations: EFA, early first active period; FA, first active period; FR, first recrudescent period; Q, quiescent period; R, resting period; Rg, regressive period; SA, second active period; SR, second recrudescent period

**Fig. 4:**
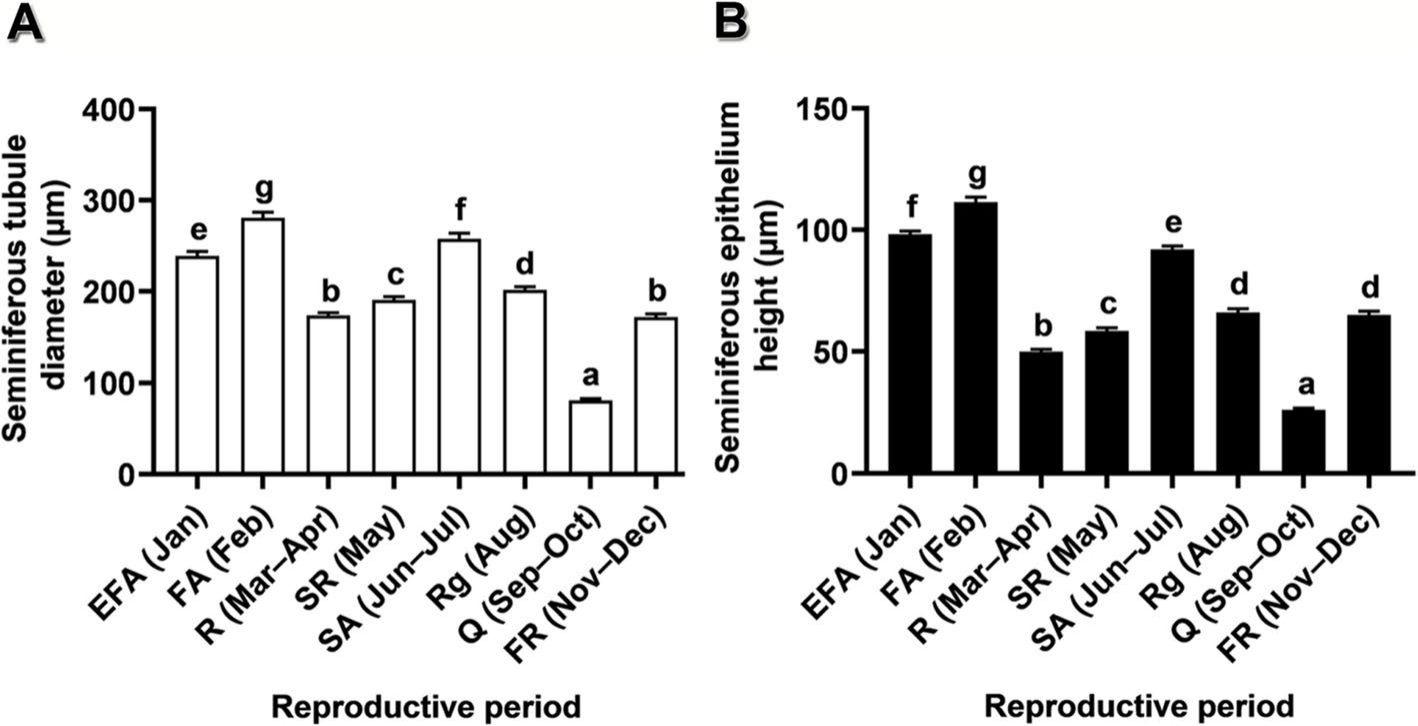
Testicular histomorphomety of *Leiolepis ocellata* during the annual reproductive cycle. **A** Seminiferous tubule diameter. **B** Seminiferous epithelium height. Each testicular histomorphometric value was obtained from 30 measurements from randomly selected histological sections of the three animals in the same reproductive periods, and presented as mean ± SE. Different letters in (**A**, **B)** denote significant differences of each histomorphometric variable among reproductive periods (*P* < 0.05)

**Fig. 5:**
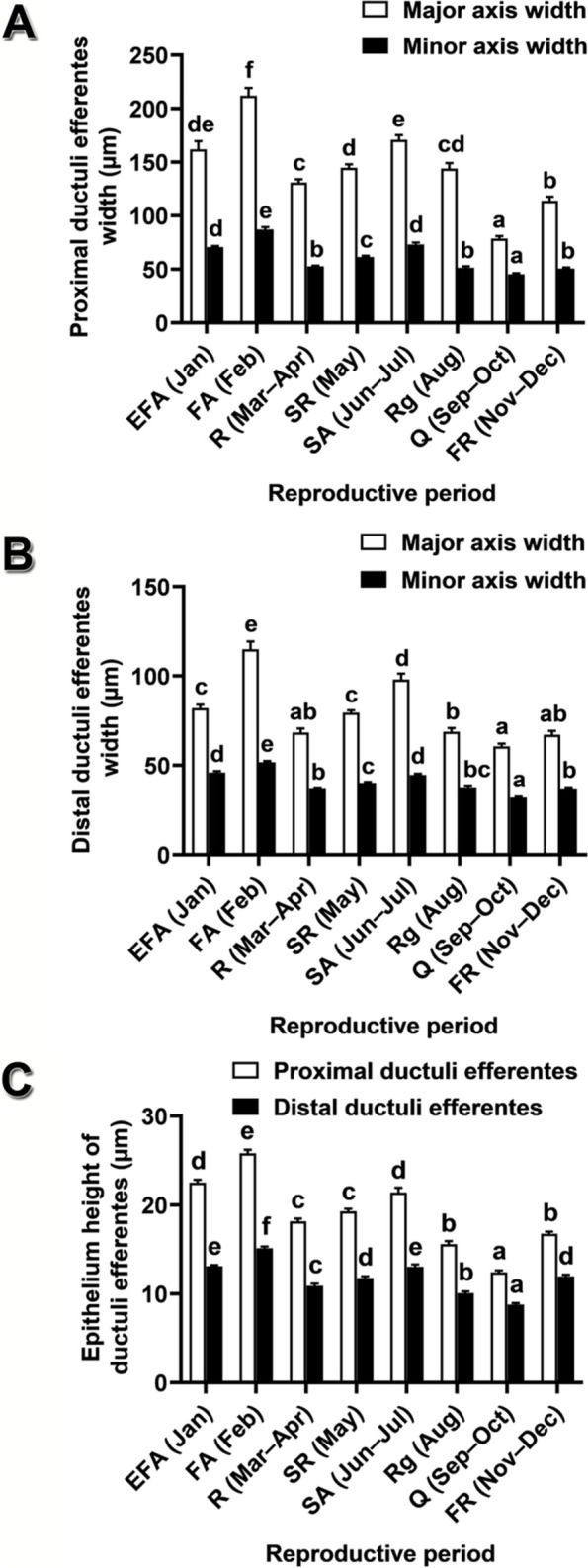
Histomorphometric characteristics of the ductuli efferentes of *Leiolepis ocellata* during the annual reproductive cycle. **A** Proximal ductuli efferentes width. **B** Distal ductuli efferentes width. **C** Epithelium height of the ductuli efferentes. Each histomorphometric value was obtained from 30 measurements from randomly selected histological sections of the three animals in the same reproductive periods, and expressed as mean ± SE. Different letters in (**A**–**C)** indicate significant differences of each histomorphometric data among reproductive periods (*P* < 0.05)

**Fig. 6:**
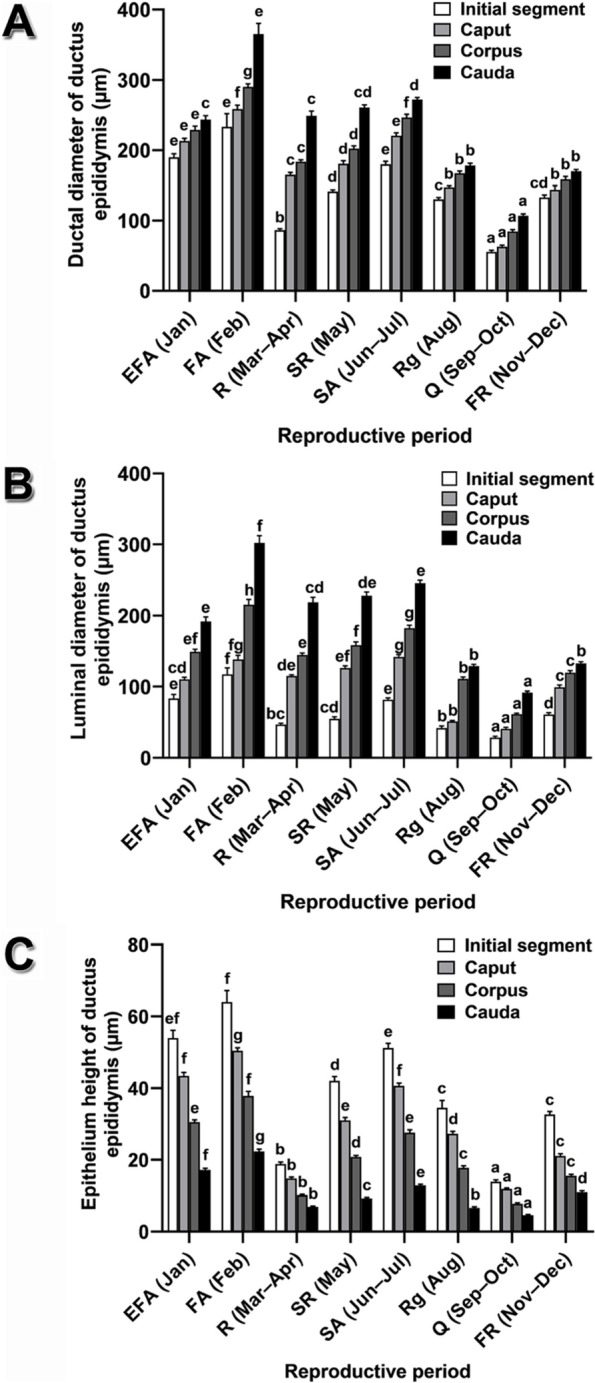
Histomorphometric characteristics of the ductus epididymis of *Leiolepis ocellata* during the annual reproductive cycle. **A** Ductal diameter of the ductus epididymis. **B** Luminal diameter of the ductus epididymis. **C** Epithelium height of the ductus epididymis. Each histomorphometric value was acquired from 30 measurements from randomly selected histological sections of the three animals in the same reproductive periods, and expressed as mean ± SE. Different letters in (**A**–**C)** denote significant differences of each histomorphometric data among reproductive periods (*P* < 0.05)

**Fig. 7:**
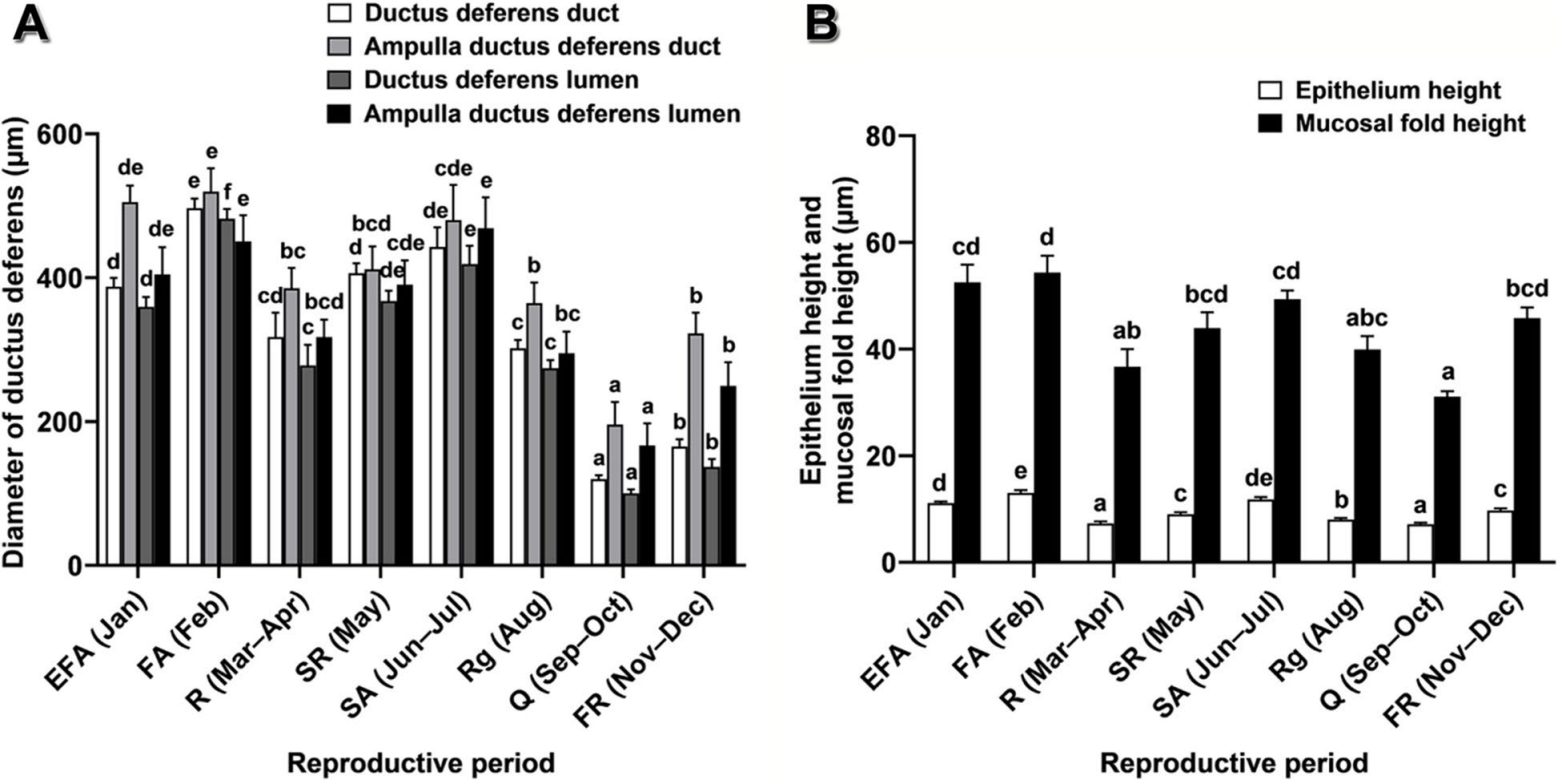
Histomorphometric characteristics of the ductus deferens of *Leiolepis ocellata* during the annual reproductive cycle. **A** Diameter of the ductus deferens. **B** Epithelium and mucosal fold heights. Each histomorphometric value was attained from 30 measurements from randomly selected histological sections of the three animals in the same reproductive periods, and presented as mean ± SE. Different letters in **A** and **B** indicate significant differences of each histomorphometric data among reproductive periods (*P* < 0.05)

The reproductive cycle of male *Leiolepis ocellata* was divisible into eight periods based on changes in histomorphological and histochemical characteristics of the reproductive structures, and sex hormonal patterns (Figs. 3, 4, 5, 6 and 7, S1, S2, S3, S4, S5, S6, S7, S8, S9 and S10). These periods consisted of (1) the early first active period (January), (2) the first active period (February), (3) the resting period (March–April), (4) the second recrudescent period (May), (5) the second active period (June–July), (6) the regressive period (August), (7) the quiescent period (September–October), and (8) the first recrudescent period (November–December). Having two active reproductive periods, male *L. ocellata*, therefore, exhibited a bimodal pattern of the reproductive cycle. Of note, a large number of spermatozoa and secretory materials were found in the lumina of the ductus deferens during the active periods (Figs. S9, S10; Table 5). In addition, juveniles were exclusively observable in the second recrudescent and the regressive periods after the first and the second active periods, respectively. These scenarios point to the possibility of mating during the first and the second active periods. In the present study, the smallest sexually mature males had a snout-vent length (SVL) of 11.70 ± 0.21 cm and had spermatozoa in the lumen of the seminiferous tubules and of the ductus epididymis.

Male reproductive structures enlarged to their full sizes in the early and the first active periods. In the resting period, the reproductive organs became reduced. Full development of the reproductive organs with their enlargement was observable again in the second active period. The reproductive organs underwent atrophy in the regressive period. During the quiescent period, the reproductive organs were regressed. Development of germ cells and genital ducts was found in the first recrudescent period. The lizards started becoming behaviorally less active in the late quiescent period and the first recrudescent period because they spent time mainly in the burrows without activities on the ground. This inactive behavior continued to the early active period of the reproductive cycle. Besides, the largest amount of fat body was detectable in the regressive and the quiescent periods, followed by its continual decline to the smallest in the first active period (Table 1). The relatively minimal amount of fat body was maintained until the second active period, while considerable amount of fat body was detectable again during the regressive period (Table 1).

**Table 1:** Morphometric characteristics of the body and the testis, and fat body quantity of *Leiolepis ocellata* (mean ± SE). Different superscript letters of the values in each column represent significant differences among each morphometric data (*P* < 0.05)

Reproductive period (month)	Sample size	Snout-vent length (cm)	Body weight (g)	Testis weight (g)		GSI	Fat body
				Left	Right		
EFA (Jan)	3	11.70 ± 0.21^a^	53.74 ± 2.18^ab^	0.19 ± 0.04^d^	0.18 ± 0.00^d^	0.36 ± 0.06^d^	+
FA (Feb)	10	13.90 ± 0.24^bcd^	68.69 ± 3.24^b^	0.27 ± 0.02^e^	0.27 ± 0.02^e^	0.35 ± 0.02^e^	+
R (Mar–Apr)	22	13.20 ± 0.26^bc^	61.59 ± 3.67^ab^	0.06 ± 0.00^b^	0.06 ± 0.00^abc^	0.11 ± 0.01^abc^	+
SR (May)	4	16.03 ± 0.48^d^	100.26 ± 11.67^c^	0.09 ± 0.01^c^	0.09 ± 0.01^bc^	0.10 ± 0.00^bc^	+
SA (Jun–Jul)	18	20.09 ± 5.63^cd^	88.44 ± 3.87^c^	0.11 ± 0.01^c^	0.11 ± 0.01^c^	0.13 ± 0.10^c^	+
Rg (Aug)	5	13.46 ± 0.30^bc^	66.37 ± 6.63^b^	0.05 ± 0.00^b^	0.05 ± 0.00^ab^	0.08 ± 0.02^ab^	+++
Q (Sep–Oct)	5	12.02 ± 0.65^a^	46.57 ± 4.53^a^	0.03 ± 0.00^a^	0.03 ± 0.00^a^	0.07 ± 0.00^a^	+++
FR (Nov–Dec)	8	12.63 ± 0.27^ab^	62.52 ± 2.94^ab^	0.05 ± 0.00^b^	0.05 ± 0.00^ab^	0.09 ± 0.02^ab^	++

Abbreviations: *+* small quantity, *++* medium quantity, *+++* large quantity, *GSI* gonadosomatic index, *SVL* snout-vent length

### Gonadosomatic index (GSI)

The left and right testes of *Leiolepis ocellata* had comparable masses in every reproductive period (Table 1). The testes were regressed during the quiescent period, showing the lowest mean paired testis mass (0.03 g) and the lowest mean GSI (0.07%) (Table 1). The mean testis mass markedly increased in the early first active period (0.19 g) and reached its peak during the first active period (0.27 g) (*P* < 0.05) (Table 1). However, both reproductive periods revealed the comparably highest GSI of approximately 0.35%. GSIs reached their highest peak in the early first active and the first active periods, while those in the other periods were relatively at the basal level. After the first active period, the mean testis masses and GSIs declined precipitously from the resting period to the regressive period although slight increment of GSI was detectable in the second active period. The increase in the testis masses coincided with phases of progressive spermatogenesis and spermiogenesis.

### Plasma sex steroid concentrations during the annual reproductive cycle

Plasma levels of T, E_2_, and P showed a similar hormonal profile during the annual reproductive cycle (Fig. 3). In general, low levels of these sex steroids were observable in the quiescent, the resting, and the regressive periods, while their maximal levels were noticeable in the first and the second active periods. Plasma T and P concentrations progressively increased from their minimal levels in the quiescent period (0.244 ± 0.004 ng/mL for T; 0.534 ± 0.387 ng/mL for P) to their first peaks in the first active period (1.503 ± 0.124 ng/mL for T; 1.590 ± 0.363 ng/mL for P) (Fig. 3A, B). In contrast, the plasma E_2_ concentration rose steeply from its basal level in the quiescent period (120.400 ± 14.456 pg/mL) to its first significantly high level in the first recrudescent period (296.000 ± 86.062 pg/mL), and remained relatively stable in the early first active period and the first active period (314.900 ± 61.290 and 311.400 ± 61.290 pg/mL, respectively) (Fig. 3C). After a sharp decrease of plasma sex steroid levels during the resting period, plasma T concentrations steadily increased (Fig. 3A), while plasma E_2_ and P concentrations escalated to their second peaks in the second active period (Fig. 3B, C). Subsequently, all sex steroids dropped significantly to the minimal levels in the regressive period.

### Histomorphological structures of the male reproductive system during the annual reproductive cycle

#### Testis

Annual changes in seminiferous tubule diameters and in seminiferous epithelium heights showed a similar trend (Fig. 4). These two parameters revealed their minimal values in the quiescent period, followed by their significantly progressive increment from the first recrudescent period to the first active period, exhibiting the maximal values (Fig. 4). After the first active period, these values significantly decreased in the resting period and significantly increased in the second recrudescent and the second active periods (Fig. 4). Subsequently, the reduced values were observable again in the regressive period (Fig. 4).

Microscopic descriptions of the male reproductive structures were based on the previous report [37]. The seminiferous tubules of the lizards in the early first active period housed primary and secondary spermatogonia, primary and secondary spermatocytes, round and elongated spermatids, and spermatozoa (Fig. S1A). The cytoplasm of Leydig cells had few and small fat vacuoles. In the first active period, numerous spermatozoa were discernible in the lumen of the seminiferous tubules (Fig. S1B). Notably, elongating/elongated spermatids and spermatozoa were more than those in the other periods. Fat vacuoles were rare in the cytoplasm of the Leydig cells. The seminiferous tubules during the resting period revealed similar histological structures to those in the quiescent period with some additional residual cells in the lumen (Fig. S1C). Sertoli cells had reduced sizes. The interstitial cells possessed many fat vacuoles and eccentric, flattened nuclei. The seminiferous epithelium during the second recrudescent period comprised primary and secondary spermatogonia, primary and secondary spermatocytes, round and elongated spermatids, and a small number of spermatozoa in the lumen (Fig. S1D). Leydig cells became fully hypertrophied and their cytoplasm had a small number of fat vacuoles. The microscopic structures of the seminiferous epithelium in the second active period were similar to those of the first active period (Fig. S1E versus S1B), but fewer germ cells, as determined by means of seminiferous tubule diameters and of seminiferous epithelium heights (Fig. 4). During the regressive period, the seminiferous epithelium was composed of primary and secondary spermatogonia, primary spermatocytes, and residual germ cells (Fig. S1F). Leydig cells contained prominent fat vacuoles. In the quiescent period, seminiferous tubules contained primary and secondary spermatogonia, and primary spermatocytes (Fig. S1G). Many fat vacuoles were accumulated in the cytoplasm of Leydig cells. In the first recrudescent period, primary and secondary spermatogonia, primary and secondary spermatocytes, and some spermatids were detectable in the seminiferous epithelium (Fig. S1H). Fewer fat vacuoles were found in the cytoplasm of Leydig cells.

#### Rete testis

The rete testis underwent annual histological variation of the lining epithelium (Table 2). During the regressive, the quiescent, and the first recrudescent periods, the rete testis was lined with a simple squamous epithelium with sparse microvilli (Fig. S2F–H; Table 2). In the other periods, the rete testis epithelium had numerous microvilli (Fig. S2A–E; Table 2). Spermatozoa were not found in the lumen during the quiescent and the first recrudescent periods (Fig. S2G, H; Table 2). Residual round germ cells were discernible in the lumen during the quiescent, the first recrudescent, and the second recrudescent periods (Fig. S2D, G, H; Table 2). The rete testis epithelium was weakly reactive to PAS during the second recrudescent and the second active periods, but not reactive to AB and BB throughout the annual reproductive cycle (Table 2).

**Table 2:** Histological and histochemical characteristics of the rete testis of *Leiolepis ocellata* during the reproductive cycle

Histological & histochemical characteristics	Reproductive periods							
	EFA (Jan)	FA (Feb)	R (Mar–Apr)	SR (May)	SA (Jun–Jul)	Rg (Aug)	Q (Sep–Oct)	FR (Nov–Dec)
Epithelium	SSq/SCu	SSq/SCu	SSq/SCu	SCu	SCu	SSq	SSq	SSq
PAS-H	–	–	–	+	+	–	–	–
AB pH 1.0	–	–	–	–	–	–	–	–
AB pH 2.5	–	–	–	–	–	–	–	–
BB	–	–	–	–	–	–	–	–
Spermatozoa	+	+	+	+	+	+	–	–
Residual round germ cells	–	–	–	+	–	–	+	+

Abbreviations: *SC* simple columnar epithelium, *SCu* simple cuboidal epithelium, *SSq* simple squamous epithelium

#### Ductuli efferentes

Ductuli efferentes are divided into the proximal and the distal regions. Widths of major and minor axes and epithelium heights of the proximal and of the distal regions exhibited histomorphological changes during the annual reproductive cycle, showing an undulated pattern (Fig. 5). These histomorphometric variables were minimal in the quiescent period, rose continuously to their highest levels in the first active period, dropped in the resting period, increased again to their second peak in the second active period and subsequently declined in the regressive period (Fig. 5). Therefore, the histological structures of the proximal and the distal ductuli efferentes during the quiescent period were less active than those in the other periods (Figs. S3G versus S3A–F, H, and S4G versus S4A–F, H). In the early first, the first active, and the second active periods, increased epithelium heights were likely attributed to the presence of a columnar epithelium (Figs. 5C, S3A, B, E; Table 3). Additionally, the epithelial cells showed more intense histochemical reactions to PAS, AB pH 1.0 and 2.5, and BB, suggesting increased production of neutral, sulfated acid and carboxylated acid polysaccharides/glycoproteins, and total proteins, respectively (Table 3). In the proximal region, apocrine blebs were absent in the quiescent period, suggesting inactive secretory activities (Fig. S3G; Table 3). Spermatozoa were exclusively present in the lumen during the active periods (Fig. S3A, B; Table 3). The distal ductuli efferentes in the quiescent period were reduced in sizes (Fig. S4G), as compared with those in the other periods (Fig. S4A–F, H).

**Table 3:** Microanatomical and histochemical characteristics of the ductuli efferentes of *Leiolepis ocellata* during the reproductive cycle

Ductuli efferentes	Histological & histochemical characteristics	Reproductive periods							
		EFA (Jan)	FA (Feb)	R (Mar–Apr)	SR (May)	SA (Jun–Jul)	Rg (Aug)	Q (Sep–Oct)	FR (Nov–Dec)
Proximal region	Epithelium	SCu/SC	PC	SCu/SC	SCu/SC	PC	PC	SCu	PC
	PAS	+	+	+	+	+	+	+	+
	AB pH 1.0	+	+	–	–	+	–	–	–
	AB pH 2.5	+	+	–	–	+	–	–	–
	BB	+	+	–	–	+	–	–	–
	Apocrine bleb	+	+	+	+	+	+	–	+
	Spermatozoa	+	+	–	–	+	–	+	–
Distal region	Epithelium	SCu/SC	SCu/SC	SCu	SCu	SCu/SC	SCu	SCu	SCu/SC
	PAS	+	+	+	+	+	+	+	+
	AB pH 1.0	–	–	–	–	–	–	–	–
	AB pH 2.5	–	–	–	–	–	–	–	–
	BB	–	–	–	–	–	–	–	–
	Apocrine bleb	–	–	–	–	–	–	–	–
	Spermatozoa	+	+	–	–	+	–	–	–

Abbreviations: *PC* pseudostratified columnar epithelium, *SC* simple columnar epithelium, SCu, simple cuboidal epithelium

#### Ductus epididymis

The ductus epididymis is divided into four regions: initial segment, caput, corpus, and cauda. Annual histological variation of all regions was found, with differences in the ductal diameter, the luminal diameter, and the epithelium height (Fig. 6). The epididymal histomorphometric parameters revealed a similar trend to those of the ductuli efferentes (Fig. 6 versus 5). All parameters had their minimal values in the quiescent period, increased progressively and reached their peaks in the first active period, declined in the resting period, rose continuously until the second active period, and decreased in the regressive period (Fig. 6). The thinnest epithelium of all epididymal regions during the quiescent period was likely due to shorter epithelial cells, as compared to those in the other reproductive periods (Figs. 6C, S5G, S6G, S7G, S8G; Table 4). Spermatozoa were found in all epididymal regions during the reproductive cycle, with the exception of the quiescent period (Figs. S5, S6, S7 and S8; Table 4). Residual germ cells were present in the epididymal lumen during the quiescent, the first recrudescent, the resting, and the second recrudescent periods (Figs. S5, S6, S7 and S8; Table 4). All epididymal regions showed histochemical reactions to PAS and BB throughout the reproductive cycle, with additional positive staining with AB pH 1.0 and 2.5 only in the quiescent period (Table 4).

**Table 4:** Microscopic structures and histochemical characteristics of the ductus epididymis of *Leiolepis ocellata* during the reproductive cycle

Ductus epididymis	Histological & histochemical characteristics	Reproductive periods							
		EFA (Jan)	FA (Feb)	R (Mar–Apr)	SR (May)	SA (Jun–Jul)	Rg (Aug)	Q (Sep–Oct)	FR (Nov–Dec)
Initial segment	Epithelium	PC	PC	PC	PC	PC	PC	PC/SCu	PC
	PAS	+	+	+	+	+	+	+	+
	AB pH 1.0	–	–	–	–	–	–	+	–
	AB pH 2.5	–	–	–	–	–	–	+	–
	BB	+	+	+	+	+	+	+	+
	Spermatozoa	+	+	+	+	+	+	–	–
	Residual round germ cells	–	–	+	+	–	–	+	+
Caput	Epithelium	PC	PC	PC	PC	PC	PC	PC/SCu	PC
	PAS	+	+	+	+	+	+	+	+
	AB pH 1.0	–	–	–	–	–	–	+	–
	AB pH 2.5	–	–	–	–	–	–	+	–
	BB	+	+	+	+	+	+	+	+
	Spermatozoa	+	+	+	+	+	+	–	–
	Residual round germ cells	–	–	+	+	–	–	+	+
Corpus	Epithelium	PC	PC	PC	PC	PC	PC	PC/SCu	PC
	PAS	+	+	+	+	+	+	+	+
	AB pH 1.0	–	–	–	–	–	–	+	–
	AB pH 2.5	–	–	–	–	–	–	+	–
	BB	+	+	+	+	+	+	+	+
	Spermatozoa	+	+	+	+	+	+	–	+
	Residual round germ cells	–	–	+	+	–	–	+	+
Cauda	Epithelium	PC	PC	PC	PC/SCu	PC	PC/SCu	SSq	SCu
	PAS	+	+	+	+	+	+	–	+
	AB pH 1.0	–	–	–	–	–	–	+	–
	AB pH 2.5	–	–	–	–	–	–	+	–
	BB	+	+	+	+	+	+	+	+
	Spermatozoa	+	+	+	+	+	+	–	+
	Residual round germ cells	–	–	+	+	–	–	+	+

Abbreviations: *PC* pseudostratified columnar epithelium, *SCu* simple cuboidal epithelium, *SSq* simple squamous epithelium

#### Ductus deferens

The ductus deferens is divided into the ductal portion and the ampulla portion (Figs. 7, S9, S10). Histomorphometric variables, comprising diameters of the duct and the lumen of both portions, revealed a similar pattern to those of the other reproductive structures during the annual reproductive cycle (Fig. 7). These variables showed the lowest values in the quiescent period, increased steadily and reached their highest values in the first active period, dropped in the resting period, escalated until the second active period, and declined in the regressive period. Annual histological variation in the epithelium types was detectable in the ductal portion (Fig. S9; Table 5). In this portion, a simple cuboidal epithelium was present throughout the annual cycle, while a pseudostratified columnar epithelium was also found in the first active and the second active periods (Fig. S9; Table 5). Conversely, the epithelium of the ampulla ductus deferens was a pseudostratified columnar epithelium throughout the cycle (Fig. S10; Table 5). PAS reactivity was detectable along the ductus deferens throughout the cycle, while BB reactivity was generally found in almost all periods, except for the quiescent and the first recrudescent periods (Table 5). None of AB-positive reactions was detectable (Table 5). Residual round germ cells were present in the lumen of the ampulla ductus deferens during the resting, the second recrudescent, the regressive, and the quiescent periods (Fig. S10C, D, F, G; Table 5). Conversely, only spermatozoa were found in the lumen of the ampulla ductus deferens during the early first active, the first active, the second active, and the first recrudescent periods (Fig. S10A, B, E, H; Table 5).

**Table 5:** Microanatomy and histochemical characteristics of the ductus deferens of *Leiolepis ocellata* during the reproductive cycle

Ductus deferens	Histological & histochemical characteristics	Reproductive periods							
		EFA (Jan)	FA (Feb)	R (Mar–Apr)	SR (May)	SA (Jun–Jul)	Rg (Aug)	Q (Sep–Oct)	FR (Nov–Dec)
Ductal portion	Epithelium	PC/SCu	SCu	SCu	SCu	SCu	SCu	SCu	PC/SCu
	PAS	+	+	+	+	+	+	+	+
	AB pH 1.0	–	–	–	–	–	–	–	–
	AB pH 2.5	–	–	–	–	–	–	–	–
	BB	+	+	+	+	+	+	–	–
	Spermatozoa	+	+	+	+	+	+	+	–
	Residual round germ cells	–	–	+	+	–	+	+	–
Ampulla portion	Epithelium	PC	PC	PC	PC	PC	PC	PC	PC
	PAS	+	+	+	+	+	+	+	+
	AB pH 1.0	–	–	–	–	–	–	–	–
	AB pH 2.5	–	–	–	–	–	–	–	–
	BB	+	+	+	+	+	+	–	–
	Spermatozoa	+	+	+	+	+	+	–	+
	Residual round germ cells	–	–	+	+	–	+	+	–

Abbreviations: *PC* pseudostratified columnar epithelium, *SCu* simple cuboidal epithelium

#### Climatic correlates of histomorphological structures of the male reproductive system

In general, three environmental parameters (temperature, rainfall, and relative humidity) were important in the regression models (Tables S1, S2, S3 and S4). Temperature and relative humidity displayed strong correlation with GSI and histomorphological structures of the male reproductive system, while rainfall did not show correlation with seminiferous tubule diameter, seminiferous epithelium height, major axis width of proximal ductuli efferentes, ductal diameter of caput epididymis, ductal diameter and epithelium height of ampulla ductus deferens (Tables S1, S2, S3 and S4). Therefore, rainfall was the physical variable of least importance in the regression model.

## Discussion

The annual reproductive cycle of male *Leiolepis ocellata* was divided into eight periods, based on changes of plasma sex steroid levels, and histomorphological and histochemical characteristics of the reproductive structures, unlike other reptiles including *Hemidactylus flaviviridis* [17] and *Ophisops elegans* [38] with three periods; *Opheodrys aestivus* [39], *Sceloporus aeneus* [27], and *S. mucronatus* [40] with four periods; *Microgecko helenae* (formerly *Tropiocolotes helenae*) with five periods [38]; and *Dipsas mikanii* (formerly *Sibynomorphus mikanii*) with six periods [31]. In addition, the reproductive cycle of *L. ocellata* exhibited a biannual/bimodal pattern of the reproductive cycle, having two peaks of the active periods, as in other reptiles, including *Agkistrodon contortrix* [41], *A. piscivorus* [32], *A. piscivorus leucostoma* [42], *Masticophis bilineatus* [43], *Sceloporus spinosus* [44], and *Vipera aspis* [45, 46]. Therefore, there existed two recrudescent periods, each preceding its corresponding active period, and the two active periods were separated by the resting (March–April) and the second recrudescent (May) periods.

In this study, concentrations of circulating sex steroids (T, E_2_, and P) were associated with testis sizes and masses, seminiferous epithelium heights, genital duct histomorphology, and histochemistry, resembling those in other reptiles with a prenuptial type of spermatogenesis [17, 47–51]. Steady concentrations of the three sex hormones during the early first active to the first active periods in *L. ocellata* may be implicated in courtship, mating, and agonistic behaviors, as suggested for *H. flaviviridis* [17]. Plasma T concentrations were increased in the male *L. ocellata* during the recrudescent period, reached the highest level in the active period, and felled thereafter, as reported in other reptiles, including *Naja kaouthia* [52], *N. naja* [53], *Podarcis siculus* [47], *Sceloporus undulatus* [18], and *Thamnophis sirtalis concinnus* [54]. In particular, increased levels of plasma T in the first recrudescent period has been suggested to be related to stimulation of spermatogonial multiplication and spermatogenesis, as described in other lizards, such as *Calotes versicolor* [55] and *Psammodromus algirus* [6]. High levels of plasma T in male *L. ocellata* during the active period were also concurrent with depletion of cytoplasmic lipid droplets in Leydig cells, whereas large quantities of lipid droplets were found during the non-active period, as in *A. contortrix*, *Liodytes pygaea* (formerly *Seminatrix pygaea*) [56], *Chrysemys picta* [57], *N. naja* [58, 59], *Natrix natrix*, *Teira dugesii* (formerly *Lacerta dugesii*), *Testudo graeca* [60], *Pelodiscus sinensis* (synonym of *Trionyx sinensis*) [61], *Phrynosoma cornutum*, and *S. mucronatus* [62]. It has been suggested that the cytoplasmic lipid droplets in Leydig cells contain precursors that are utilized for androgen biosynthesis [57, 63], also involved in reproductive behaviors [62], spermatogenesis progress, and spermiogenesis completion [64]. Plasma T is also essential for transformation of round spermatids to elongated spermatids during spermiogenesis [65], sperm production, reproductive behaviors (territoriality, courtship, and copulation) [51, 55, 66, 67], secretory activities of the male genital ducts [68] and of the sexual segments of the kidney [66, 69–71], and secondary sexual characteristics [72]. Therefore, plasma T in *L. ocellata* likely plays roles in spermatogenesis, spermiogenesis, and development of male genital ducts.

Profiles of plasma E_2_ concentrations in male *L. ocellata* were similar to those of plasma T during the early first active, the first active and the second active periods, as observed in *H. flaviviridis* [17] and *Tiliqua nigrolutea* [19]. Plasma E_2_ may be responsible for spermiogenesis by directly binding to estrogen receptors on spermatids and spermatozoa for maintaining and preventing spermatid death [73]. Several studies have reported that E_2_ in male reptiles may influence reproductive behaviors [19, 74, 75] and testicular growth [17]. Contrarily, E_2_ is at low levels during the active period of the snake *V. aspis* [76] and the lizard *P. s. siculus* [47]. It has been suggested that E_2_ suppresses spermatogenic activity of *P. siculus* [77].

Plasma P concentrations in male *L. ocellata* were correlated with the reproductive events. It began to increase during spermatogenesis in the recrudescent period, reached the highest level at the final stage of gamete maturation in the active period, and decreased sharply after peaks of spermatogenesis, as previously described [78]. It has been suggested that P can bind directly to progesterone receptors on germ cells in all stages of development [20], likely implicated in sexual maturation and stimulation of male reproductive behaviors [19, 20, 79], as shown in the lizard, *Aspidoscelis inornatus* (synonym of *Cnemidophorus inornatus*) [80, 81]. In mammals, P can influence spermiogenesis and sperm capacitation [82]. It is known that P is important as a substrate for T and E_2_ biosynthesis [83]. This accounts for positive correlation between the circulating levels of P and T/E_2_. Conversely, exogenously-administered P exerts suppressive effects on male sexual behaviors in the lizard *Anolis carolinensis* [21].

*Leiolepis ocellata* became behaviorally inactive and they spent approximately four months (late October to January) of the cold season in the burrows without outdoor activities. This duration and characteristics for inactive behavior were similar to those during hibernation of the snake *V. aspis* [45] and the lizard *Acanthodactylus schreiberi syriacus* [84]. At this time, the reproductive organs underwent recrudescence. It has been suggested that the hibernation period is important for germ cell renewal [85]. In male *L. ocelllata*, spermatogenesis occurred during this behaviorally inactive period, with primary and secondary spermatocytes in the seminiferous epithelia. Therefore, male *L. ocelllata* displayed prenuptial spermatogenic pattern, in which spermatogenesis occurs before copulation, as seen in *Barisia imbricata* [86], *Marisora brachypoda* (synonym of *Mabuya brachypoda*) [87], and *Trachylepis capensis* (formerly *Mabuya capensis*) [88]. Thereafter, spermatozoa were found in the seminiferous tubules at post-hibernation, similar to other reptiles, such as *C. picta* [89], *O. elegans* [38], *P. algirus* [6], and *Trapelus ruderatus* (formerly *Trapelus lessonae*) [90].

Male lizards reserve energy as stored fat bodies, which are utilized on purposes during their reproductive cycle, such as winter nutrition, testicular recrudescence, male-male competition, territory defense, searching for females, and courtship behaviors [11, 91–94]. In *L. ocellata*, the amount of fat body was related to gonad and genital duct histomorphology and to plasma sex hormone levels, suggesting that the fat body may be a source of energy used during the period of behavioral inactivity, spermatogenesis, and sex steroid hormone production*,* as proposed for *Liolaemus bitaeniatus* [95] and *Scincus mitranus* [96] that have large fat bodies during the quiescent period with abundant food, and show depleted fat bodies during spermiogenesis. Increased fat storage reflects decreased energy expenditure for reproductive activities [97]. The significantly higher GSIs in the early first active/first active periods than those in the second active period are likely attributed to both the higher testis masses and the lower body masses in the former as a result of stored fat utilization during behavioral inactivity in the first recrudescent period.

In the resting and the second recrudescent periods, spermatozoa were found in the lumen of the caput, the corpus, and the cauda ductus epididymis, and the ductus deferens, while spermiogenesis was absent and the epithelia of these genital ducts were not actively secretory, suggesting that male *L. ocellata* stored spermatozoa produced previously from the first active period for the second mating season. Supporting this hypothesis, reptilian sperm storage has been shown in different regions of the male genital ducts, viz. the ductus epididymis, as seen in the snake, *A. piscivorus* [32] and the freshwater turtles, *C. picta* and *Trachemys scripta* [98]; the ductus deferens, as observed in the snake, *Crotalus durissus terrificus* [99], the snakes of the family Colubridae [100] and the caiman, *Caiman crocodilus* [101]; and both the ductus epididymis and the ductus deferens, as in the lizards, *Sitana ponticeriana* [102] and *L. ocellata* in the present study. In particular, the snake, *A. piscivorus leucostoma*, has been proposed to display two breeding seasons, with the first copulatory period in late summer/fall and the second mating period in spring [42].

The testis sizes and microanatomy of *L. ocellata* underwent annual cyclical changes, similar to other lizards [28, 103]. Nonetheless, the left and right testes of *L. ocellata* had almost equivalent masses throughout the reproductive periods, similar to those in the lizards *Tenuidactylus caspius* (formerly *Cyrtopodion caspium*) [104], *Hoplodactylus* spp., and *Naultinus* spp. [105], suggesting that the paired testes of *L. ocellata* had comparable reproductive activities. In addition, changes of the microscopic structures of *L. ocellata* testes were correlated with reproductive periods of the reproductive cycle, as previously described in other species [1].

The lowest values for seminiferous tubule diameters and seminiferous epithelium heights were found in the quiescent period, similar to *A. piscivorus* [32]. Hypertrophy of Sertoli cells was associated with the reproductive periods, with them becoming fully hypertrophied in the first and the second active periods, while undergoing regression in the other periods, similar to the lizard *Eutropis carinata* (synonym of *Mabuya carinata*) [106].

Spermatogonia A and B were present in the seminiferous epithelium in every reproductive period, suggesting that they serve as a stockpile of germ cell production. In the quiescent period, there were only these pre-meiotic cells in the seminiferous epithelium, as in *A. carolinensis* [107], *A. piscivorus* [32], *Hemidactylus turcicus* [108], *S. aeneus* [27], *S. mucronatus* [40], *S. spinosus*, [109], and *T. scripta* [110]. Primary and secondary spermatocytes were present during the recrudescent period, similar to the lizards, *Anolis porcatus* [111], *S. spinosus* [109], and *S. variabilis* [112], and the snakes, *D. mikanii* [31], *Masticophis taeniatus*, and *Pituophis melanoleucus* [113]. Spermatids and spermatozoa were the predominant cell types during the active periods, suggesting active spermiogenesis, as in other lizards [17, 26, 27, 114, 115]. In the resting period, the seminiferous epithelium and spermiogenesis were less active, but spermatozoa and residual germ cells were found in the lumina of the seminiferous tubules, the ductus epididymis, and the ductus deferens, as previously described [27, 31, 40].

Male genital ducts of *L. ocellata* underwent histomorphological and histochemical variation during the annual reproductive cycle. This variation included epithelium heights, ductal and luminal diameters, and secretory activities throughout the year, similar to the previous reports [30, 116], and was related to functional dynamics of the seminiferous epithelium (i.e., spermatogenesis and spermiogenesis), testis masses, diameters of the seminiferous tubules, and sex steroid hormone levels. Notably, the male genital ducts are controlled by sex steroid hormones [117]. The presence of two peaks of spermatogenesis, in conjunction with two peaks of active histomorphology of the male genital ducts, is similar to those found in *H. turcicus* [118]. During the quiescent period, the ductuli efferentes showed weak staining with PAS, suggesting that their secretions contained neutral carbohydrates and might be responsible for lubricating the ductal lumen [32]. The ductuli efferentes was lined with pseudostratified cuboidal/columnar epithelia, with the exception of those in the quiescent period, resembling those in *S. ponticeriana* [119], but different from *A. carolinensis*, *S. undulatus* [30], and *L. pygaea* [120], with a simple cuboidal epithelium. The epithelium of the proximal and the distal ductuli efferentes revealed histochemical reactions with PAS in all periods, similar to those in *A. piscivorus* [32] and *P. platurus* [121]. Besides, different epithelium heights were observable in the ductuli efferentes, as in *P. platurus* [121]. Spermatozoa were found in the lumen of the proximal and the distal ductuli efferentes during the early first active, the first active, and the second active periods, similar to *A. piscivorus* [32].

Histological changes of the ductus epididymis during the reproductive cycle were correlated with development of the seminiferous epithelium and plasma sex steroid hormone levels, as previously described [68, 122]. In addition, the staining patterns of the luminal secretory granules in the corpus and the cauda epididymis from the early first active period to the regressive period were similar to those of *A. piscivorus* [32] and *H. turcicus* [118]. Positive staining of the secretory granules with PAS and BB in *L. ocellta* is suggested that these granules contained glycoproteins, similar to *A. piscivorus* [32], *L. pygaea* [120], *P. platurus* [121], and *T. elegans* [123].

The ductal portion of the ductus deferens had a relatively uniform shape and type of the epithelium in all reproductive periods, but different epithelium heights. Furthermore, changes of the ductal diameter were found during the reproductive cycle, as seen in *A. piscivorus* [32]. During the resting and the second active periods, the epithelium became thinner to accommodate sperm storage before ejaculation. In addition, masses of spermatozoa were found in the lumen during the resting and the second recrudescent periods, suggesting that *L. ocellata* may store spermatozoa for the second mating. Spermatozoa were found in the lumen of the ampulla portion during the resting and second recrudescent periods, like the ductal portion, suggesting that they were the site for sperm storage. Therefore, both ductal and ampulla portions likely served as a principal site for sperm storage. Less secretory activity of the ductus deferens in *L. ocellata* in the course of the reproductive cycle was similar to that found in *A. piscivorus* [32]. In the present study, the ductal and the ampulla ductus deferens are likely responsible for secretory activity for sperm storage during the resting and the second recrudescent periods.

In this study, environmental factors were correlated with GSIs and histomorphological variables in the regression models. It has been shown that the environmental cues, such as temperature, rainfall, and photoperiod, are significant exogenous stimuli controlling male reproductive activities in several lizards that have seasonal reproductive cycles [22, 86, 124–128]. High temperatures may serve as a predictor for aridity and lead to less energetic investment for reproduction in lizards, thus retarding reproductive activities [129]. This may account for inactive reproduction of *L. ocellata* in the resting period, especially in April that had the highest temperature. Herein, copulation of *L. ocellata* possibly took place twice during the annual reproductive cycle, namely in the first active period (February) and perhaps in the early resting period (March), and the second active period (June), with subsequent occurrence of juveniles in the second recrudescent period (May of early monsoon season) and the regressive period (August of mid-monsoon season), respectively. Supporting this postulation, there existed a copious number of spermatozoa in the ductus deferens of *L. ocellata* during the first and the second active periods, thus readily preparing for insemination. The two periods for emergence of the juveniles in the monsoon season may be related to rainfall that influences vegetation, thus resulting in abundant food supply for the young, like plant shoots and insects. Taken together, it is suggested that coordination between climatic factors and reproductive activities allows for suitable resources essential for successful generation and survival of newly-emerging progeny [33, 130, 131]. Besides, the presence of large fat bodies in the lizards during the regressive and quiescent periods (August–September) suggests that they prepare energy reserve for their residence in the burrows during late October–January. Therefore, biannual reproduction of male *L. ocellata* may be considered as a plastic response to favorable environmental factors, namely an adaptation for reproductive success.

## Conclusion

This article describes variation of histomorphometric, histochemical and hormonal profiles of the male reproductive system of *L. ocellata* during the annual reproductive cycle. The reproductive cycle of male *L. ocellata* was divisible into eight periods with a bimodal pattern. Plasma E_2_, P and T levels were related to histomorphometric and histochemical profiles of the male reproductive organs, with their highest concentrations during the active period, coinciding with full hypertrophy of the testes and the genital ducts and active secretory functions, but low hormonal levels during the inactive periods with reduced reproductive structures. Plasma T was likely associated with germ cell development, and hypertrophy and secretory activities of the male genital ducts. Circulating E_2_ was suggested to be involved in spermiogenesis, while plasma P may participate in sperm maturation.

The highest histomorphometric values of the reproductive structures were noticeable in the first and the second active periods in conjunction with peaks of circulating sex hormones. Leydig cells underwent cyclic changes with the cytoplasm containing many fat vacuoles during the nonbreeding period, but fewer cytoplasmic vacuoles in the active period. Abundance of male germ cells is related to hypertrophy of the genital ducts.

Three environment factors (temperature, rainfall, and relative humidity) showed correlation with GSI and histomorphometric characteristics of the testes and the genital ducts during the annual reproductive cycle. Therefore, male *L. ocellata* seem to adapt their reproductive cycle to suit the environmental conditions that are associated with provision of food for their newborns.

## Methods

### Climatic parameters, sample collection, and determination of reproductive cycle of male *Leiolepis ocellata*

The study areas were at Tak (17°16′53.5″N, 99°08′43.9″E) and Lampang (17°33′55.0″N, 99°12′14.1″E) provinces in northern Thailand (Fig. 1). The areas (ca. 170 m above sea level) consisted of two adjacent habitats: cassava plantation and deciduous forest. Online data on the climatic parameters of the study areas over three years (2015–2017) were obtained from Bang Na Agromet Meteorological Station, Thai Meteorological Department.

A total of 75 adult male *Leiolepis ocellata* individuals were collected monthly from September 2015 to August 2016 (Table 1). Species identification was based on previous reports [35, 132, 133]. The animals were captured using a traditional snare trap. It is noted that the animals started becoming behaviorally less active in late October and they spent their time mostly in the burrows. The inactive behavior continued to January. Therefore, the animals were collected during this time by only excavating them from their burrows instead of using the trap. After sample collection, they were immediately transported to the laboratory at Department of Zoology, Kasetsart University in Bangkok, maintained overnight in paper boxes and fed with insects. Blood and reproductive tissues were collected around 9 a.m. on the following day.

Intraperitoneal injection of sodium pentobarbital (1 μL/g of body mass) to the animals was performed to induce anesthesia. The snout-vent length (SVL) was measured using a measuring tape to the nearest mm and the body mass was determined using a digital scale to the nearest 0.01 g. Subsequently, midventral abdominal incision from the cloacal orifice towards the thoracic region was made to expose internal organs. Blood from the carotid artery was collected using a 1 mL syringe with a 24-guage needle, transferred into heparinized tubes and centrifuged at 100×g (10 min, 4 °C). The plasma was collected and stored at − 80 °C for further sex steroid analysis. The testes and genital ducts were removed from the animals for microanatomical and histochemical study. The testis mass was determined using a digital scale. Gonadosomatic index (GSI) was calculated using the formula: GSI = [testis mass (g)/body mass (g)] × 100. The amount of fat body was evaluated and given the scores as small (+), medium (++) and large (+++) quantities.

Criteria for determination of the male reproductive cycle of *L. ocellata* were not justifiable on the basis of gross appearance of the animals. Herein, our classification of the reproductive cycle was based on changes in histomorphological and histochemical characteristics of the reproductive system, and sex hormonal profiles.

### Histomorphometric and histochemical study

The testes and genital ducts were preserved in Bouin’s fixative. Thereafter, tissues were dehydrated using an ethanol series with increasing concentrations, cleared in xylene and embedded in Paraplast Plus® (Sigma-Aldrich, USA). The tissues were sectioned into 5 μm-thick slices and attached onto gelatin-coated glass slides. Histological sections were stained with hematoxylin and eosin (H&E) for nuclear and cytoplasmic visualization, Masson’s trichrome for demonstration of muscular and collagenous tissues, periodic acid-Schiff and haematoxylin (PAS–H) for neutral glycoproteins/mucopolysaccharides, alcian blue (AB) 8GX pH 1.0 and 2.5 for sulfated acid and carboxylated acid glycoproteins/mucopolysaccharides, respectively, and nuclear fast red (NR) as a nuclear counterstaining dye for AB (AB–NR), and bromophenol blue (BB) for general proteins [134]. Microscope slides were viewed under a bright-field microscope (Olympus BX51) and photomicrographs were taken using a digital camera (Olympus DP70 Camera System).

Following photomicrography, histomorphometric analyses of the reproductive tissues from the animals of different reproductive periods were performed using the Image J software (version 1.50 g; Wayne Rasban Software Design: National Institutes of Health, USA). Histomorphometric variables comprised seminiferous tubule diameters, seminiferous epithelium heights, diameters of major and minor axes of proximal and distal ductuli efferentes, epithelium heights of proximal and distal ductuli efferentes, ductal and luminal diameters of ductus epididymis (initial segment, caput, corpus and cauda), epithelium heights of ductus epididymis (initial segment, caput, corpus and cauda), ductal and luminal diameters of ductus deferens (ductal and ampulla ductus deferens), epithelium heights of ductus deferens and mucosal fold heights of ampulla ductus deferens. Each histomorphometric value was obtained from 30 measurements from randomly selected histological sections of the three representative specimens in the same reproductive periods, and expressed as mean ± SE.

### Plasma sex steroid assay

Plasma sex steroid concentrations (estradiol (E_2_), progesterone (P) and testosterone (T)) were measured. Four individuals from each reproductive period were randomly selected for hormonal analysis, with the exception in January having three specimens. The total blood plasma from each individual was divided into three portions for quantification of the three hormones. One hundred microliters of each plasma portion were mixed with 2 mL of diethyl ether via vortexing for 30 sec. The mixture was centrifuged at 1600×g for 5 min. The upper liquid ether phase was collected into a 1.5 mL microcentrifuge tube. The ether was evaporated under nitrogen gas stream at room temperature, and the dried steroids were desiccated and stored at − 80 °C until hormonal analysis. Plasma P and T concentrations were quantified using chemiluminescence immunoassay (CMIA) (Vet Cal Laboratory Center at Nontri Pet Hospital). Sensitivity of CMIA for P and T assay was 0.46 ng/mL and 0.01 ng/mL, respectively. Plasma E_2_ concentrations were measured using commercially available enzyme-linked immunosorbent assay (ELISA) kit (Cayman Chemical, USA, #582251). Sensitivity of ELISA assay for E_2_ was 15 pg/mL. All procedures for plasma E_2_ assay were performed according to manufacturer instructions. All hormonal quantification was conducted in duplicates and the average values were used.

### Statistical analysis

SPSS version 23 was used to analyze data. Data were tested for normality using Komolgorov-Smirnov test and homogeneity of variances using Levene’s test. The Box Cox transformation was performed (log, reciprocal and power transformations), when necessary, to satisfy the assumptions for parametric tests. One-way analysis of variance (ANOVA) with reproductive periods as the factor was used to examine whether hormonal, morphometric and histomorphometric variables revealed annual changes during the reproductive cycle. Multiple comparisons were conducted using Duncan’s or Games-Howell post hoc tests to determine statistical differences. However, non-transformed data were used for data presentation and graphical illustration for simplicity of data visualization. Results were presented as means ± SE, and *P* values of less than 0.05 were considered statistically significant. Multiple regressions were used to test for correlation of three environmental factors (temperature, rainfall, and relative humidity) with GSI and histomorphometric parameters as the dependent variables.

## Supplementary Information

**Additional file 1.**

**Additional file 2.**

## Data Availability

The materials of *Leiolepis ocellata* (embedded paraffin blocks, paraffin sections and stained histological slides) are stored at Department of Zoology, Faculty of Science, Kasetsart University. Raw data collected and all images taken are available from the corresponding author on reasonable request.

## Abbreviations

AB: Alcian blue
AB–NR: Alcian blue-neutral red
BB: Bromophenol blue
E_2_: Estradiol
EFA: Early first active period
ES: Elongating and elongated spermatid
FA: First active period
FR: First recrudescent period
FV: Fat vacuole
GSI: Gonadosomatic index
H&E: Hematoxylin and eosin
LC: Leydig cell
MF: Mucosal fold
P: Progesterone
PAS: Periodic acid-Schiff
PAS-H: Periodic acid-Schiff-hematoxylin
PC: Pseudostratified columnar epithelium
Pi: Pigment cell
PS: Primary spermatocyte
Q: Quiescent period
R: Resting period
RC: Residual round germ cell
Rg: Regressive period
RS: Round spermatid
SA: Second active period
SC: Simple columnar epithelium
SCu: Simple cuboidal epithelium
Se: Sertoli cell
Sg: Spermatogonium
SG: Secretory granule
SS: Secondary spermatocyte
SSq: Simple squamous epithelium
SVL: Snout-vent length
SR: Second recrudescent period
Sz: Spermatozoa
T: Testosterone
