# Supplementary material for: Male reproductive cycle in a population of the endemic butterfly lizard, Leiolepis ocellata Peters, 1971 (Squamata: Agamidae) from northern Thailand
Source: BMC Zool. 2022 Aug 5;7:45. doi: 10.1186/s40850-022-00145-6 (PMC10127433; doi:10.1186/s40850-022-00145-6)
Supplement: Supplementary file 1 — Additional file 1. [file 40850_2022_145_MOESM1_ESM.docx]

**Male reproductive cycle in a population of the endemic butterfly lizard, *Leiolepis ocellata* Peters, 1971 (Squamata: Agamidae) from northern Thailand**

Akkanee Pewhom^1^, Thidaporn Supapakorn^2^ and Nopparat Srakaew^3,*^

^1^Department of Biology, Faculty of Science, Thaksin University, Phatthalung, 93210, Thailand

^2^Department of Statistics, Faculty of Science, Kasetsart University, Bangkok, 10900, Thailand

^3^Department of Zoology, Faculty of Science, Kasetsart University, Bangkok, 10900, Thailand

*****Correspondence: Nopparat Srakaew (E-mail: fscinrsr@ku.ac.th)

**Additional file 1.** Supplementary tables of correlations between structures of the testes and of the genital ducts of the butterfly lizard *Leiolepis ocellata* and environmental cues in the study area.

**Table S1** Multiple regression showing correlation between the testicular structures of *Leiolepis ocellata* and environmental cues in the study area. *T*-test for the null hypothesis with *β* = 0 and significant results at *α* = 0.05.

| **Model** | **Effect** | **Estimate** | **Standard error** | ***t*** | ***P*** |
| --- | --- | --- | --- | --- | --- |
| GSI | Intercept | 2.6130 | 0.1869 | 13.98 | <0.0001 |
|  | Rainfall | 0.0011 | 0.0002 | 4.92 | <0.0001 |
|  | Humidity | -0.0139 | 0.0015 | -9.27 | <0.0001 |
|  | Temperature | -0.0559 | 0.0038 | -14.61 | <0.0001 |
|  | Overall model: *F* = 92.98, *r* = 0.8915, *P* < 0.0001 | | | | |
| Seminiferous tubule diameter | Intercept | 776.9000 | 83.0600 | 9.35 | <0.0001 |
|  | Rainfall | 0.0704 | 0.1002 | 0.70 | 0.4830 |
|  | Humidity | -3.1550 | 0.6526 | -4.84 | <0.0001 |
|  | Temperature | -12.9400 | 1.7850 | -7.25 | <0.0001 |
|  | Overall model: *F* = 33.14, *r* = 0.5444, *P* < 0.0001 | | | | |
| Seminiferous epithelium height | Intercept | 404.7000 | 30.1700 | 13.41 | <0.0001 |
|  | Rainfall | 0.0638 | 0.0364 | 1.75 | 0.0812 |
|  | Humidity | -1.5310 | 0.2371 | -6.46 | <0.0001 |
|  | Temperature | -8.2000 | 0.6486 | -12.64 | <0.0001 |
|  | Overall model: *F* = 82.11, *r* = 0.7146, *P* < 0.0001 | | | | |

**Table S2** Multiple regression showing correlation between the histomorphological structures of ductuli efferentes of *Leiolepis ocellata* and environmental factors in the study area. *T*-test for the null hypothesis with *β* = 0 and significant results at *α* = 0.05.

| **Model** | **Effect** | **Estimate** | **Standard error** | ***t*** | ***P*** |
| --- | --- | --- | --- | --- | --- |
| Proximal ductuli efferentes width (major axis) | Intercept | 573.0000 | 61.4200 | 9.33 | <0.0001 |
|  | Rainfall | 0.0605 | 0.0762 | 0.79 | 0.4278 |
|  | Humidity | -2.6700 | 0.4873 | -5.48 | <0.0001 |
|  | Temperature | -8.9770 | 1.3300 | -6.75 | <0.0001 |
|  | Overall model: *F* = 32.60, *r* = 0.5582, *P* < 0.0001 | | | | |
| Proximal ductuli efferentes width (minor axis) | Intercept | 288.10 | 18.19 | 15.83 | <0.0001 |
|  | Rainfall | 0.1461 | 0.0220 | 6.66 | <0.0001 |
|  | Humidity | -1.4810 | 0.1430 | -10.36 | <0.0001 |
|  | Temperature | -4.8120 | 0.3911 | -12.30 | <0.0001 |
|  | Overall model: *F* = 58.29, *r* = 0.6524, *P* < 0.0001 | | | | |
| Distal ductuli efferentes width (major axis) | Intercept | 318.2 | 30.67 | 10.38 | <0.0001 |
|  | Rainfall | 0.1598 | 0.0370 | 4.32 | <0.0001 |
|  | Humidity | -1.5620 | 0.2410 | -6.48 | <0.0001 |
|  | Temperature | -5.0700 | 0.6592 | -7.69 | <0.0001 |
|  | Overall model: *F* = 22.27, *r* = 0.4698, *P* < 0.0001 | | | | |
| Distal ductuli efferentes width (minor axis) | Intercept | 138.70 | 8.13 | 17.06 | <0.0001 |
|  | Rainfall | 0.0484 | 0.0098 | 4.93 | <0.0001 |
|  | Humidity | -0.6070 | 0.0639 | -9.50 | <0.0001 |
|  | Temperature | -2.1260 | 0.1747 | -12.16 | <0.0001 |
|  | Overall model: *F* = 63.34, *r* = 0.6679, *P* < 0.0001 | | | | |
| Proximal ductuli efferentes (epithelium height) | Intercept | 82.7200 | 4.8920 | 16.91 | <0.0001 |
|  | Rainfall | 0.0280 | 0.0059 | 4.71 | <0.0001 |
|  | Humidity | -0.4255 | 0.0388 | -10.96 | <0.0001 |
|  | Temperature | -1.3050 | 0.1059 | -12.32 | <0.0001 |
|  | Overall model: *F* = 80.47, *r* = 0.7187, *P* < 0.0001 | | | | |
| Distal ductuli efferentes (epithelium height) | Intercept | 36.5700 | 2.6180 | 13.97 | <0.0001 |
|  | Rainfall | 0.0065 | 0.0032 | 2.07 | 0.0399 |
|  | Humidity | -0.1440 | 0.0206 | -7.00 | <0.0001 |
|  | Temperature | -0.5429 | 0.0563 | -9.65 | <0.0001 |
|  | Overall model: *F* = 51.12, *r* = 0.6276, *P* < 0.0001 | | | | |

**Table S3** Multiple regression showing correlation between the histomorphological structures of ductus epididymis of *Leiolepis ocellata* and environmental parameters in the study area. *T*-test for the null hypothesis with *β* = 0 and significant results at *α* = 0.05.

| **Model** | **Effect** | **Estimate** | **Standard error** | ***t*** | ***P*** |
| --- | --- | --- | --- | --- | --- |
| Initial segment (ductal diameter) | Intercept | 850.9000 | 86.8700 | 9.80 | <0.0001 |
|  | Rainfall | 0.3559 | 0.1061 | 3.35 | 0.0010 |
|  | Humidity | -3.5470 | 0.6583 | -5.39 | <0.0001 |
|  | Temperature | -17.0400 | 1.9060 | -8.94 | <0.0001 |
|  | Overall model: *F* = 29.84, *r* = 0.6038, *P* < 0.0001 | | | | |
| Caput (ductal diameter) | Intercept | 848.40 | 95.37 | 8.90 | <0.0001 |
|  | Rainfall | 0.1841 | 0.1209 | 1.52 | 0.1298 |
|  | Humidity | -4.4720 | 0.7858 | -5.69 | <0.0001 |
|  | Temperature | -13.5600 | 1.9900 | -6.81 | <0.0001 |
|  | Overall model: *F* = 33.59, *r* = 0.6391, *P* < 0.0001 | | | | |
| Corpus (ductal diameter) | Intercept | 940.1 | 92.31 | 10.18 | <0.0001 |
|  | Rainfall | 0.2858 | 0.1114 | 2.57 | 0.0112 |
|  | Humidity | -5.1130 | 0.7252 | -7.05 | <0.0001 |
|  | Temperature | -14.8100 | 1.9840 | -7.47 | <0.0001 |
|  | Overall model: *F* = 34.25, *r* = 0.6302, *P* < 0.0001 | | | | |
| Cauda (ductal diameter) | Intercept | 1157.0000 | 113.8000 | 10.17 | <0.0001 |
|  | Rainfall | 0.4255 | 0.1373 | 3.10 | 0.0023 |
|  | Humidity | -7.6520 | 0.8942 | 8.56 | <0.0001 |
|  | Temperature | -15.6900 | 2.4460 | 6.41 | <0.0001 |
|  | Overall model: *F* = 42.66, *r* = 0.6713, *P* < 0.0001 | | | | |
| Initial segment (luminal diameter) | Intercept | 469.8000 | 45.0600 | 10.43 | <0.0001 |
|  | Rainfall | 0.2045 | 0.0542 | 3.78 | 0.0002 |
|  | Humidity | -2.1980 | 0.3384 | -6.49 | <0.0001 |
|  | Temperature | -9.4290 | 0.9682 | -9.74 | <0.0001 |
|  | Overall model: *F* = 35.88, *r* = 0.6160, *P* < 0.0001 | | | | |
| Caput (luminal diameter) | Intercept | 526.8000 | 66.3700 | 7.94 | <0.0001 |
|  | Rainfall | 0.3261 | 0.0841 | 3.88 | 0.0002 |
|  | Humidity | -3.9170 | 0.5468 | -7.16 | <0.0001 |
|  | Temperature | -6.6150 | 1.3850 | -4.78 | <0.0001 |
|  | Overall model: *F* = 24.65, *r* = 0.5799, *P* < 0.0001 | | | | |

**Table S3** Continued.

| **Model** | **Effect** | **Estimate** | **Standard error** | ***t*** | ***P*** |
| --- | --- | --- | --- | --- | --- |
| Corpus (luminal diameter) | Intercept | 570.7000 | 64.3500 | 8.87 | <0.0001 |
|  | Rainfall | 0.1703 | 0.0830 | 2.05 | 0.0418 |
|  | Humidity | -3.5150 | 0.5206 | -6.75 | <0.0001 |
|  | Temperature | -7.2820 | 1.3910 | -5.24 | <0.0001 |
|  | Overall model: *F* = 27.39, *r* = 0.5642, *P* < 0.0001 | | | | |
| Cauda (luminal diameter) | Intercept | 1100.0000 | 104.8000 | 10.50 | <0.0001 |
|  | Rainfall | 0.5657 | 0.1308 | 4.32 | <0.0001 |
|  | Humidity | -7.9110 | 0.8384 | -9.44 | <0.0001 |
|  | Temperature | -14.6900 | 2.2540 | -6.52 | <0.0001 |
|  | Overall model: *F* = 45.14, *r* = 0.6703, *P* < 0.0001 | | | | |
| Initial segment (epithelium height) | Intercept | 262.6000 | 23.5800 | 11.13 | <0.0001 |
|  | Rainfall | 0.1307 | 0.0292 | 4.47 | <0.0001 |
|  | Humidity | -1.1990 | 0.1852 | -6.48 | <0.0001 |
|  | Temperature | -5.2790 | 0.5073 | -10.41 | <0.0001 |
|  | Overall model: *F* = 39.61, *r* = 0.5870, *P* < 0.0001 | | | | |
| Caput (epithelium height) | Intercept | 226.8000 | 15.8600 | 14.30 | <0.0001 |
|  | Rainfall | 0.1102 | 0.0191 | 5.76 | <0.0001 |
|  | Humidity | -1.1080 | 0.1246 | -8.89 | <0.0001 |
|  | Temperature | -4.5410 | 0.3409 | -13.32 | <0.0001 |
|  | Overall model: *F* = 66.20, *r* = 0.6760, *P* < 0.0001 | | | | |
| Corpus (epithelium height) | Intercept | 173.5000 | 11.2400 | 15.43 | <0.0001 |
|  | Rainfall | 0.0943 | 0.0140 | 6.73 | <0.0001 |
|  | Humidity | -0.8464 | 0.0883 | -9.59 | <0.0001 |
|  | Temperature | -3.5480 | 0.2419 | -14.67 | <0.0001 |
|  | Overall model: *F* = 77.12, *r* = 0.7128, *P* < 0.0001 | | | | |
| Cauda (epithelium height) | Intercept | 103.0000 | 5.4830 | 18.78 | <0.0001 |
|  | Rainfall | 0.0298 | 0.0066 | 4.50 | <0.0001 |
|  | Humidity | -0.4844 | 0.0431 | -11.24 | <0.0001 |
|  | Temperature | -2.1360 | 0.1179 | -18.12 | <0.0001 |
|  | Overall model: *F* = 152.3, *r* = 0.8120, *P* < 0.0001 | | | | |

**Table S4** Multiple regression showing correlation between the histomorphological structures of ductus deferens of *Leiolepis ocellata* and environmental factors in the study area. *T*-test for the null hypothesis with *β* = 0 and significant results at *α* = 0.05.

| **Model** | **Effect** | **Estimate** | **Standard error** | ***t*** | ***P*** |
| --- | --- | --- | --- | --- | --- |
| Ductal ductus deferens (ductal diameter) | Intercept | 1921.0000 | 309.6000 | 6.21 | <0.0001 |
|  | Rainfall | 1.1600 | 0.3735 | 3.11 | 0.0027 |
|  | Humidity | -13.2900 | 2.4320 | -5.46 | <0.0001 |
|  | Temperature | -27.6300 | 6.6550 | -4.15 | <0.0001 |
|  | Overall model: *F* = 12.34, *r* = 0.5723, *P* < 0.0001 | | | | |
| Ampulla ductus deferens (ductal diameter) | Intercept | 1765.0000 | 340.1000 | 5.19 | <0.0001 |
|  | Rainfall | 0.5556 | 0.4102 | 1.35 | 0.1796 |
|  | Humidity | -9.3930 | 2.6720 | -3.52 | 0.0007 |
|  | Temperature | -27.2500 | 7.3100 | -3.73 | 0.0004 |
|  | Overall model: *F* = 8.24, *r* = 0.4954, *P* < 0.0001 | | | | |
| Ductal ductus deferens (luminal diameter) | Intercept | 1958.0000 | 307.0000 | 6.38 | <0.0001 |
|  | Rainfall | 1.2090 | 0.3704 | 3.27 | 0.0016 |
|  | Humidity | -13.3400 | 2.4120 | -5.53 | <0.0001 |
|  | Temperature | -29.8400 | 6.5990 | -4.52 | <0.0001 |
|  | Overall model: *F* = 12.47, *r* = 0.5744, *P* < 0.0001 | | | | |
| Ampulla ductus deferens (luminal diameter) | Intercept | 1597.0000 | 362.1000 | 4.4090 | <0.0001 |
|  | Rainfall | 0.9367 | 0.4368 | 2.1440 | 0.0352 |
|  | Humidity | -9.7550 | 2.8450 | -3.4290 | 0.0010 |
|  | Temperature | -23.4300 | 7.7840 | -3.0100 | 0.0035 |
|  | Overall model: *F* = 4.72, *r* = 0.3964, *P* < 0.01 | | | | |
| Ductal ductus deferens (epithelium height) | Intercept | 37.5200 | 4.2890 | 8.75 | <0.0001 |
|  | Rainfall | 0.0133 | 0.0052 | 2.58 | 0.0109 |
|  | Humidity | -0.1347 | 0.0337 | -4.00 | <0.0001 |
|  | Temperature | -0.6882 | 0.0922 | -7.47 | <0.0001 |
|  | Overall model: *F* = 22.13, *r* = 0.5464, *P* < 0.0001 | | | | |
| Ampulla ductus deferens (epithelium height) | Intercept | 137.3000 | 24.3800 | 5.63 | <0.0001 |
|  | Rainfall | 0.0180 | 0.0294 | 0.61 | 0.5413 |
|  | Humidity | -0.4337 | 0.1916 | -2.26 | 0.0249 |
|  | Temperature | -2.2720 | 0.5241 | -4.34 | <0.0001 |
|  | Overall model: *F* = 9.67, *r* = 0.3960, *P* < 0.0001 | | | | |
