# Supplementary material for: Male reproductive cycle in a population of the endemic butterfly lizard, Leiolepis ocellata Peters, 1971 (Squamata: Agamidae) from northern Thailand
Source: BMC Zool. 2022 Aug 5;7:45. doi: 10.1186/s40850-022-00145-6 (PMC10127433; doi:10.1186/s40850-022-00145-6)
Supplement: Supplementary file 2 — Additional file 2. [file 40850_2022_145_MOESM2_ESM.docx]

**Male reproductive cycle in a population of the endemic butterfly lizard, *Leiolepis ocellata* Peters, 1971 (Squamata: Agamidae) from northern Thailand**

Akkanee Pewhom^1^, Thidaporn Supapakorn^2^ and Nopparat Srakaew^3,*^

^1^Department of Biology, Faculty of Science, Thaksin University, Phatthalung, 93210, Thailand

^2^Department of Statistics, Faculty of Science, Kasetsart University, Bangkok, 10900, Thailand

^3^Department of Zoology, Faculty of Science, Kasetsart University, Bangkok, 10900, Thailand

*****Correspondence: Nopparat Srakaew (E-mail: fscinrsr@ku.ac.th)

**Additional file 2.** Supplementary figures of microanatomy of the testes and male genital ducts of the butterfly lizard, *Leiolepis ocellata*.

**Figure legends**

**Fig. S1** Microscopic structures of the testes at different reproductive periods. **A** Early first active period (EFA). **B** First active period (FA). **C** Resting period (R). **D** Second recrudescent period (SR). **E** Second active period (SA). **F** Regressive period (Rg). **G** Quiescent period (Q). **H** First recrudescent period (FR). Photomicrographs are specified with their corresponding reproductive period at the top-right corner. Abbreviations: ES, elongating and elongated spermatid; FV, fat vacuoles; LC, Leydig cell; PS, primary spermatocyte; RC, residual round germ cell; RS, round spermatid; Se, Sertoli cell; Sg, spermatogonium; SS, secondary spermatocyte; Sz, spermatozoa. Stains: A = PAS−H; B, D, G, H = H&E; C, E, F = Masson’s trichrome.

**Fig. S2** Microanatomical structures of the rete testis of *Leiolepis ocellata* during the annual reproductive cycle. **A** Early first active period (EFA). **B** First active period (FA). **C** Resting period (R). **D** Second recrudescent period (SR). **E** Second active period (SA). **F** Regressive period (Rg). **G** Quiescent period (Q). **H** First recrudescent period (FR). Photomicrographs are designated with their corresponding reproductive periods at the top-right corner. Abbreviations: RC, residual round germ cell; Sz, spermatozoa. Stains: A = PAS−H; B, C, G = H&E; D, F, H = Masson’s trichrome; E = AB pH 2.5–NR.

**Fig. S3** Microanatomy of the proximal ductuli efferentes of *Leiolepis ocellata* during the annual reproductive cycle. Reproductive periods are indicated at the top-right corner of their corresponding photomicrographs. Abbreviations: EFA, early first active period; FA, first active period; FR, first recrudescent period; Pi, pigment cell; Q, quiescent period; R, resting period; Rg, regressive period; SA, second active period; SR, second recrudescent period; Sz, spermatozoa. Stains: A, G = PAS−H; B = BB; C, E = AB pH 1.0–NR; D = H&E; F, H = Masson’s trichrome.

**Fig. S4** Histological structures of the distal ductuli efferentes of *Leiolepis ocellata* during the annual reproductive cycle. Micrographs are labeled at the top-right corner with their corresponding reproductive periods. Abbreviations: EFA, early first active period; FA, first active period; FR, first recrudescent period; Q, quiescent period; R, resting period; Rg, regressive period; SA, second active period; SR, second recrudescent period; Sz, spermatozoa. Stains: A, D, E = Masson’s trichrome; B, C, G = BB; F, H = PAS−H.

**Fig. S5** Histology of the initial segment of the epididymis of *Leiolepis ocellata* during the annual reproductive cycle. Photomicrographs are indicated at the top-right corner with their corresponding reproductive periods. Abbreviations: EFA, early first active period; FA, first active period; FR, first recrudescent period; Q, quiescent period; R, resting period; RC, residual round germ cell; Rg, regressive period; SA, second active period; SR, second recrudescent period; Sz, spermatozoa. Stains: A, C, E = Masson’s trichrome; B, G = BB; D = H&E; F = PAS−H; H = AB pH 1.0–NR.

**Fig. S6** Microscopic structures of the caput epididymis of *Leiolepis ocellata* during the annual reproductive cycle. Photomicrographs are labeled at the top-right corner with their corresponding reproductive periods. Abbreviations: EFA, early first active period; FA, first active period; FR, first recrudescent period; Q, quiescent period; R, resting period; Rg, regressive period; RC, residual round germ cell; SA, second active period; SR, second recrudescent period; Sz, spermatozoa. Stains: A, D, G = PAS−H; B, E, F = Masson’s trichrome; C = AB pH 1.0–NR; H = BB.

**Fig. S7** Histological structures of the corpus epididymis of *Leiolepis ocellata* during the annual reproductive cycle. Reproductive periods are specified at the top-right corner of their corresponding micrographs. Abbreviations: EFA, early first active period; FA, first active period; FR, first recrudescent period; Q, quiescent period; R, resting period; Rg, regressive period; RC, residual round germ cell; SA, second active period; SG, secretory granule; SR, second recrudescent period; Sz, spermatozoa. Stains: A, G = PAS−H; B = BB; C, D, E, H = AB pH 1.0–NR; F = Masson’s trichrome.

**Fig. S8** Microanatomical structures of the cauda epididymis of *Leiolepis ocellata* during the annual reproductive cycle. Photomicrographs are designated at the top-right corner with their corresponding reproductive periods. Abbreviations: EFA, early first active period; FA, first active period; FR, first recrudescent period; Q, quiescent period; R, resting period; Rg, regressive period; RC, residual round germ cell; SA, second active period; SG, secretory granule; SR, second recrudescent period; Sz, spermatozoa. Stains: A, F = Masson’s trichrome; B, C, D, E, G = PAS−H; H = BB.

**Fig. S9** Microanatomy of the ductal portion of the ductus deferens during the annual reproductive cycle. Reproductive periods are shown at the top-right corner of their corresponding photomicrographs. Abbreviations: EFA, early first active period; FA, first active period; FR, first recrudescent period; Q, quiescent period; R, resting period; RC, residual round germ cell; Rg, regressive period; SA, second active period; SG, secretory granule; SR, second recrudescent period; Sz, spermatozoa. Stains: A, B, C, H = H&E; D = BB; E, F, G = PAS−H.

**Fig. S10** Histology of the ampulla portion of the ductus deferens during the annual reproductive cycle. Photomicrographs are specified with their corresponding reproductive periods at the top-right corner. Abbreviations: EFA, early first active period; FA, first active period; FR, first recrudescent period; MF, mucosal fold; Q, quiescent period; R, resting period; RC, residual round germ cell; Rg, regressive period; SA, second active period; SG, secretory granule; SR, second recrudescent period; Sz, spermatozoa. Stains: A, H = PAS−H; B, G = H&E; C, E = AB pH 2.5–NR; D = AB pH 1.0–NR; F = Masson’s trichrome.


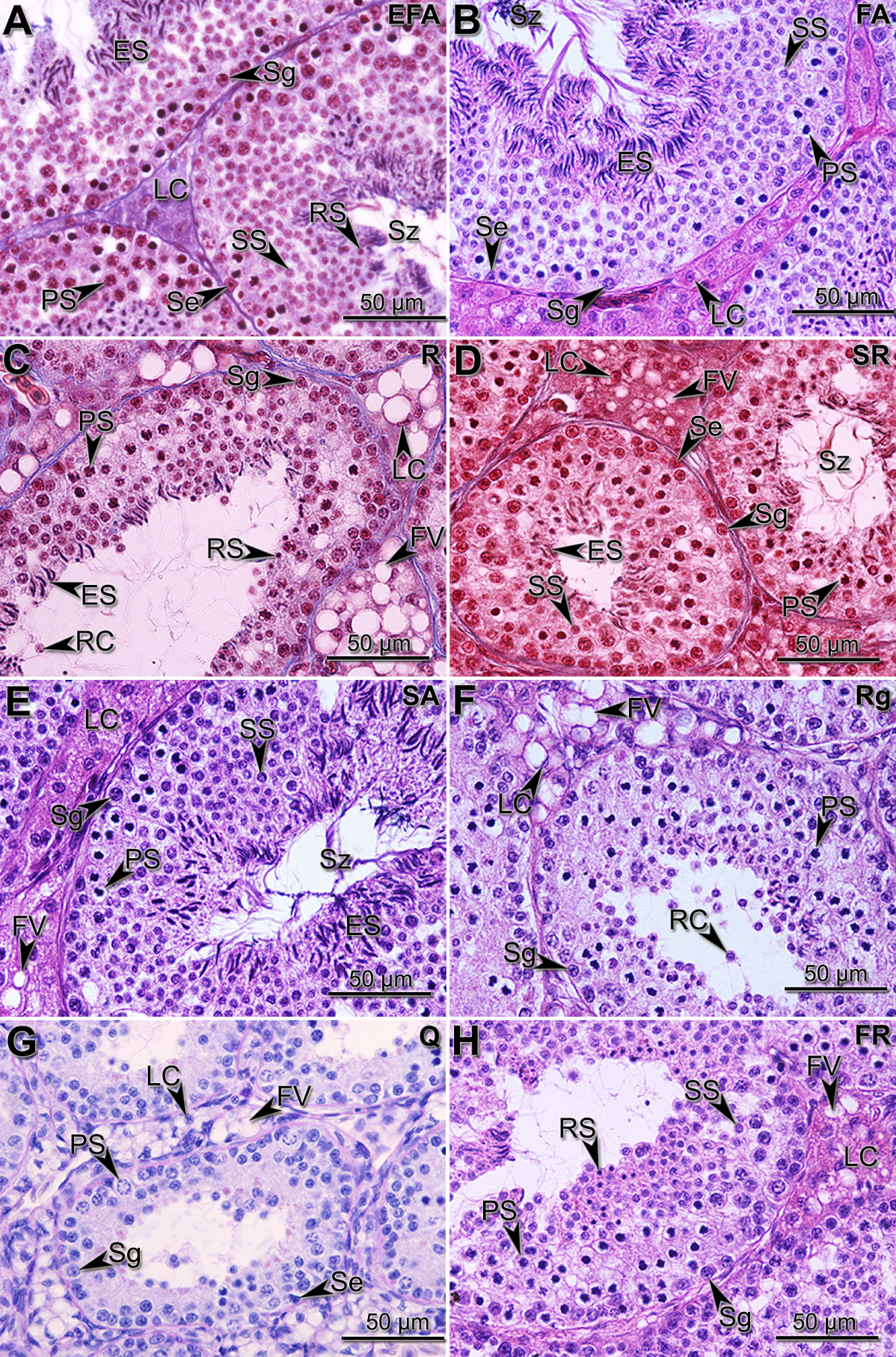


**Fig. S1**

**
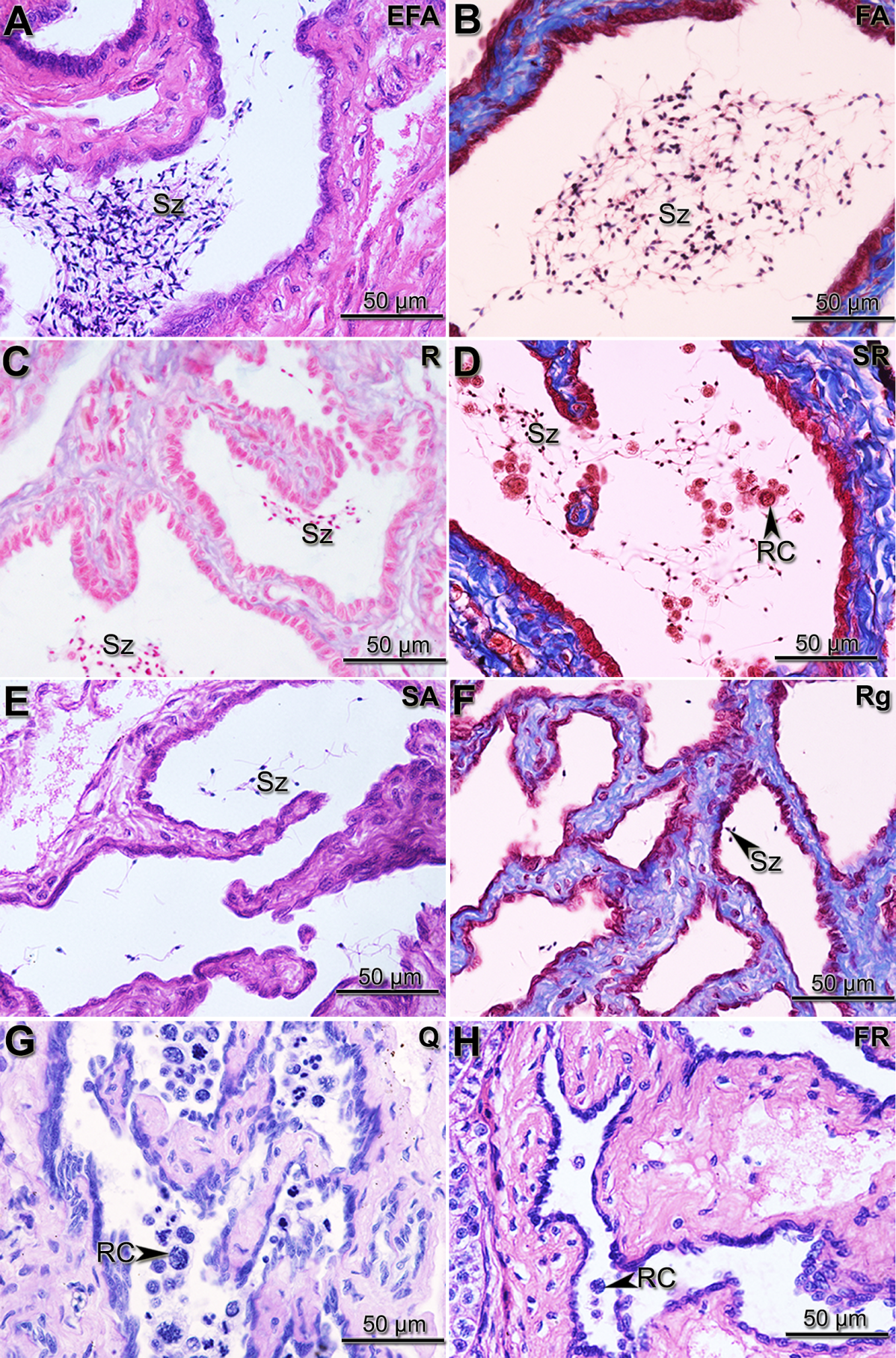
**

**Fig. S2**


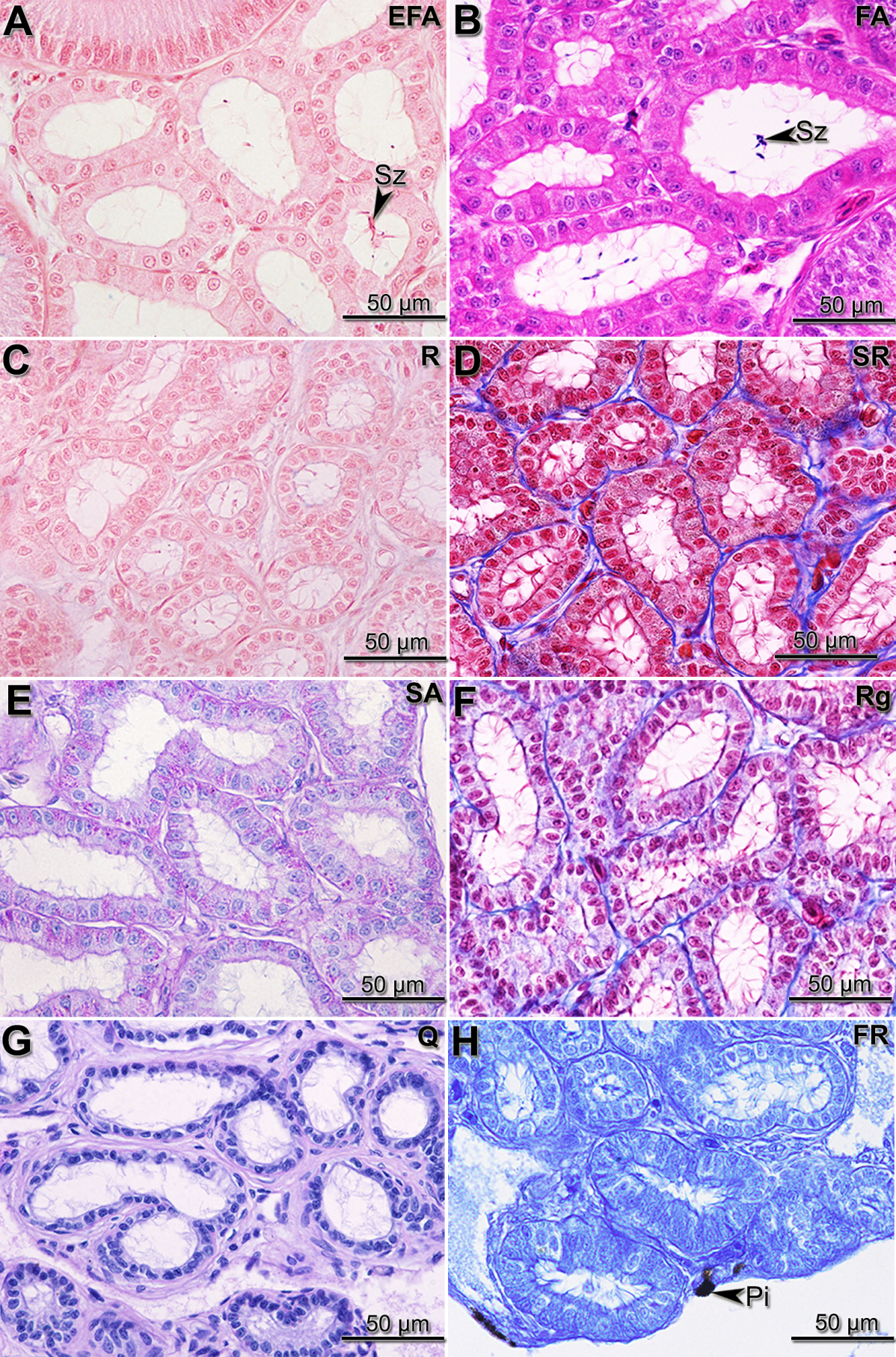


**Fig. S3**


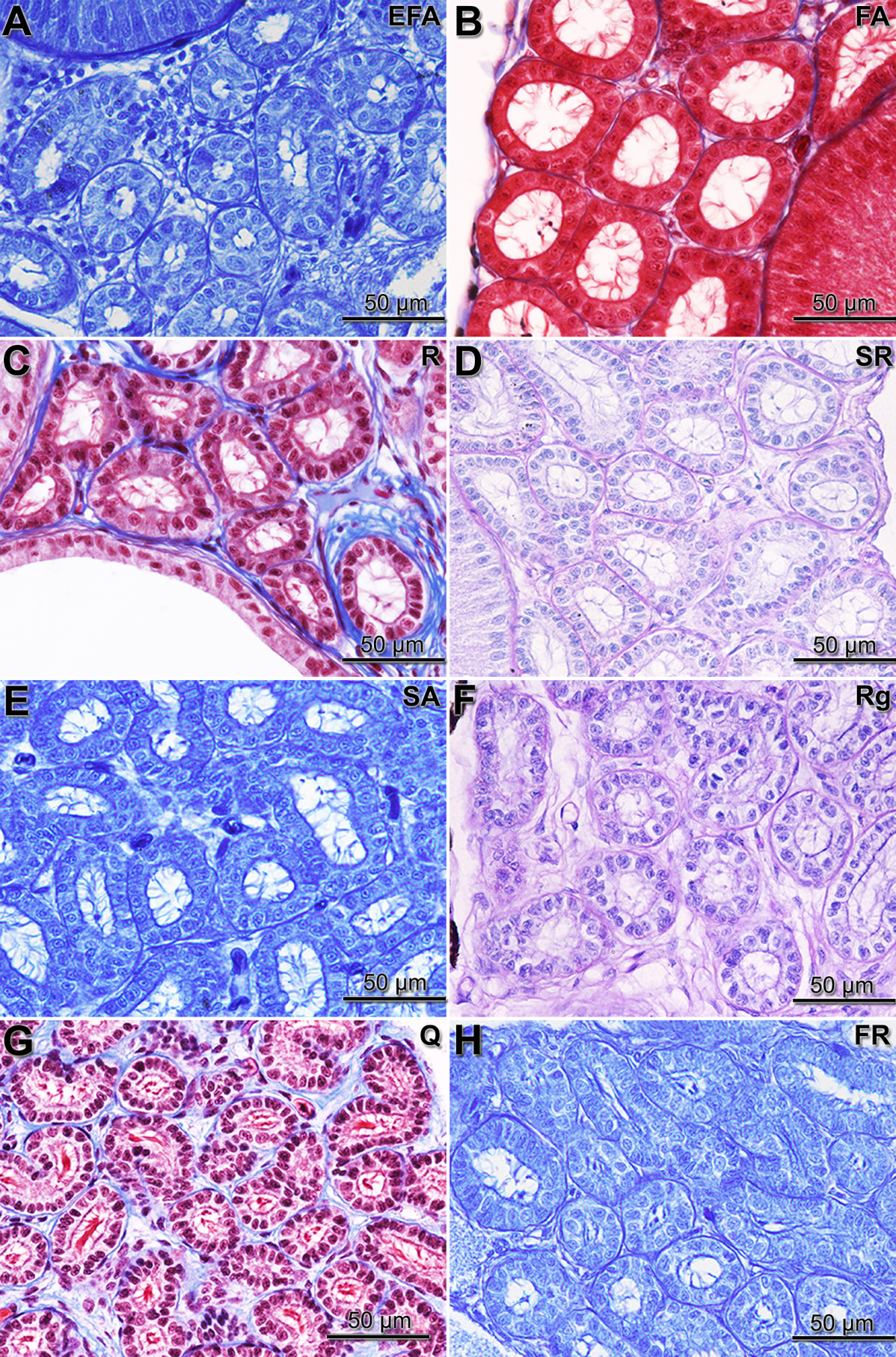


**Fig. S4**


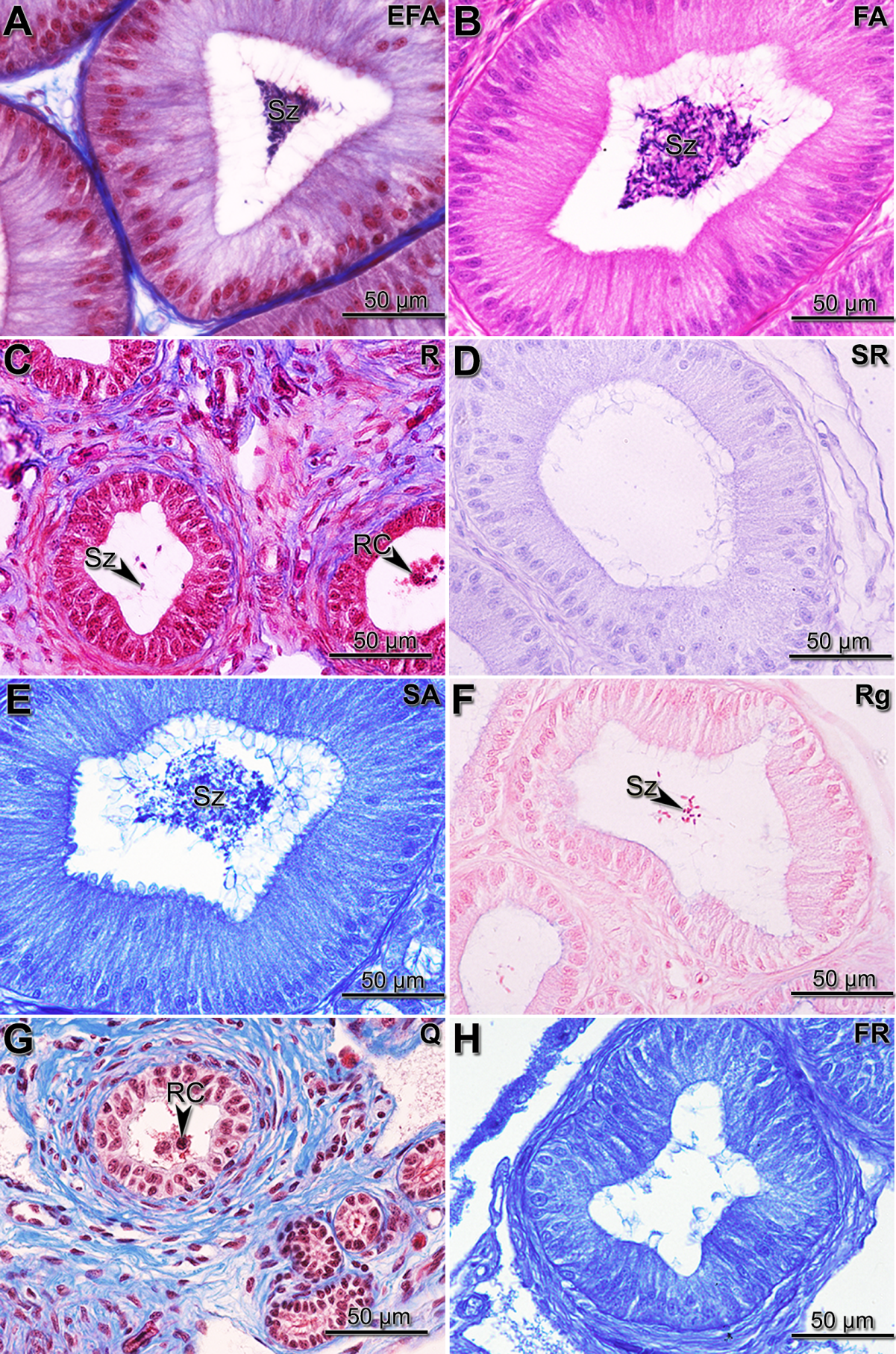


**Fig. S5**


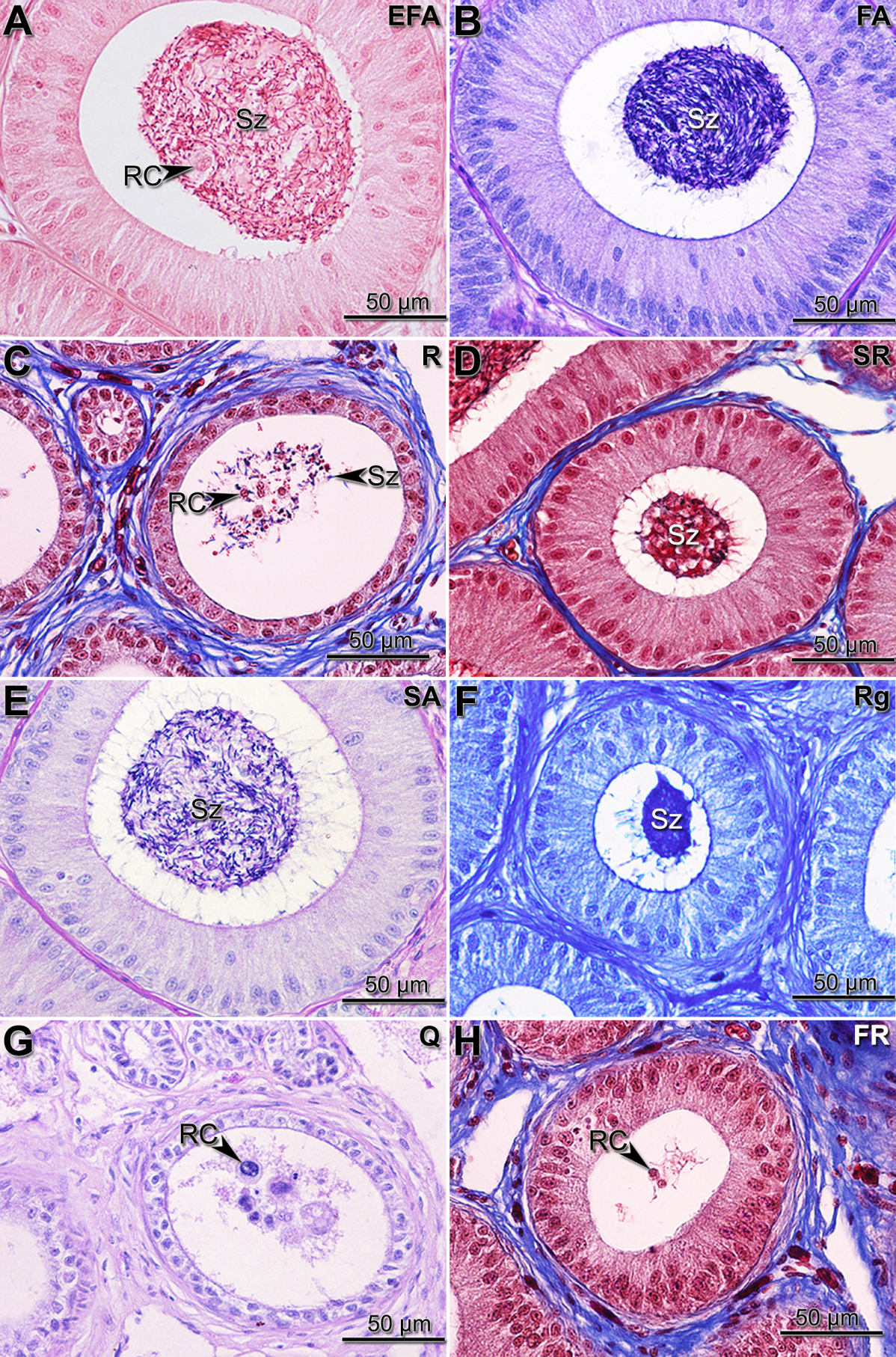


**Fig. S6**


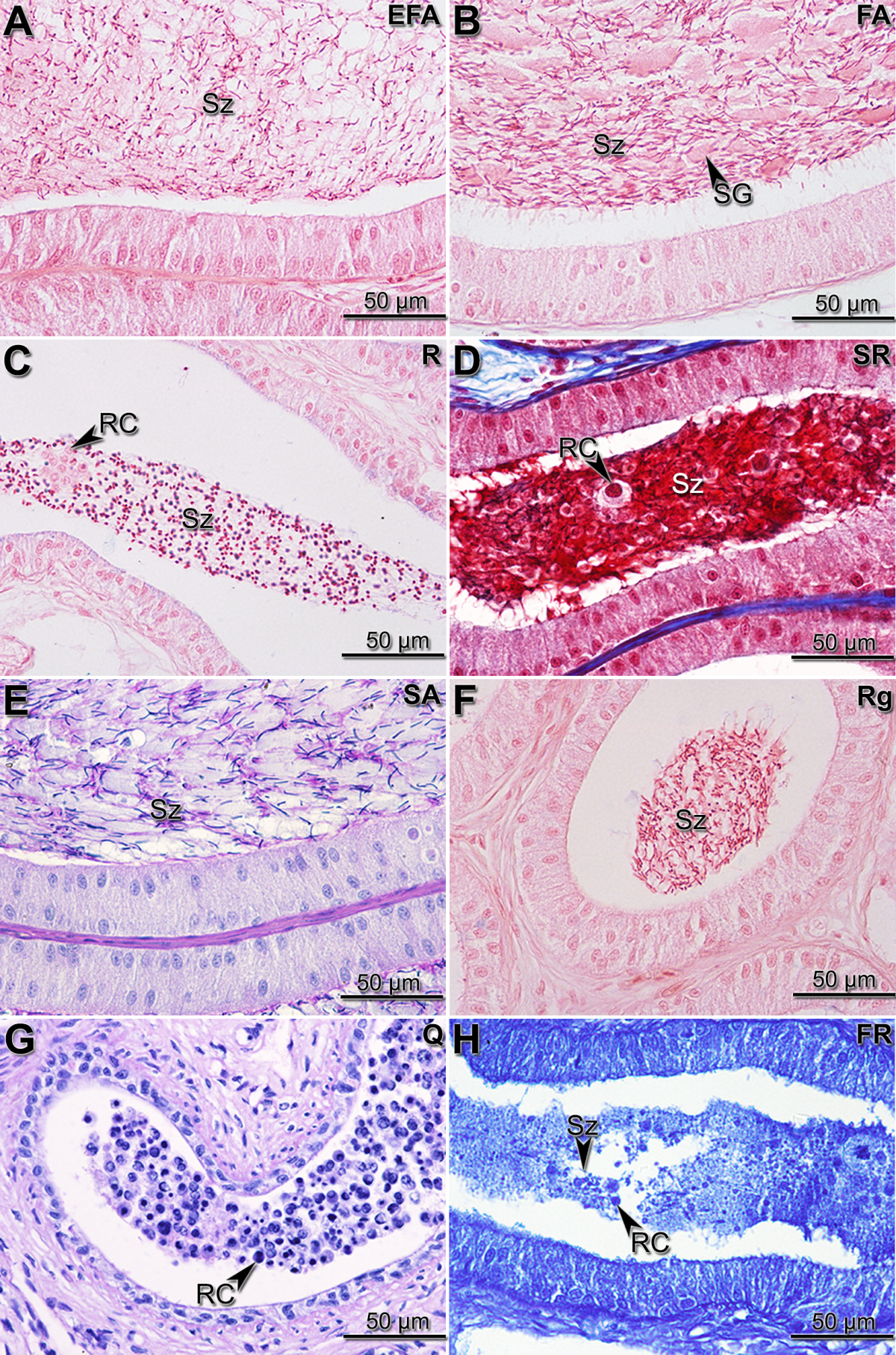


**Fig. S7**


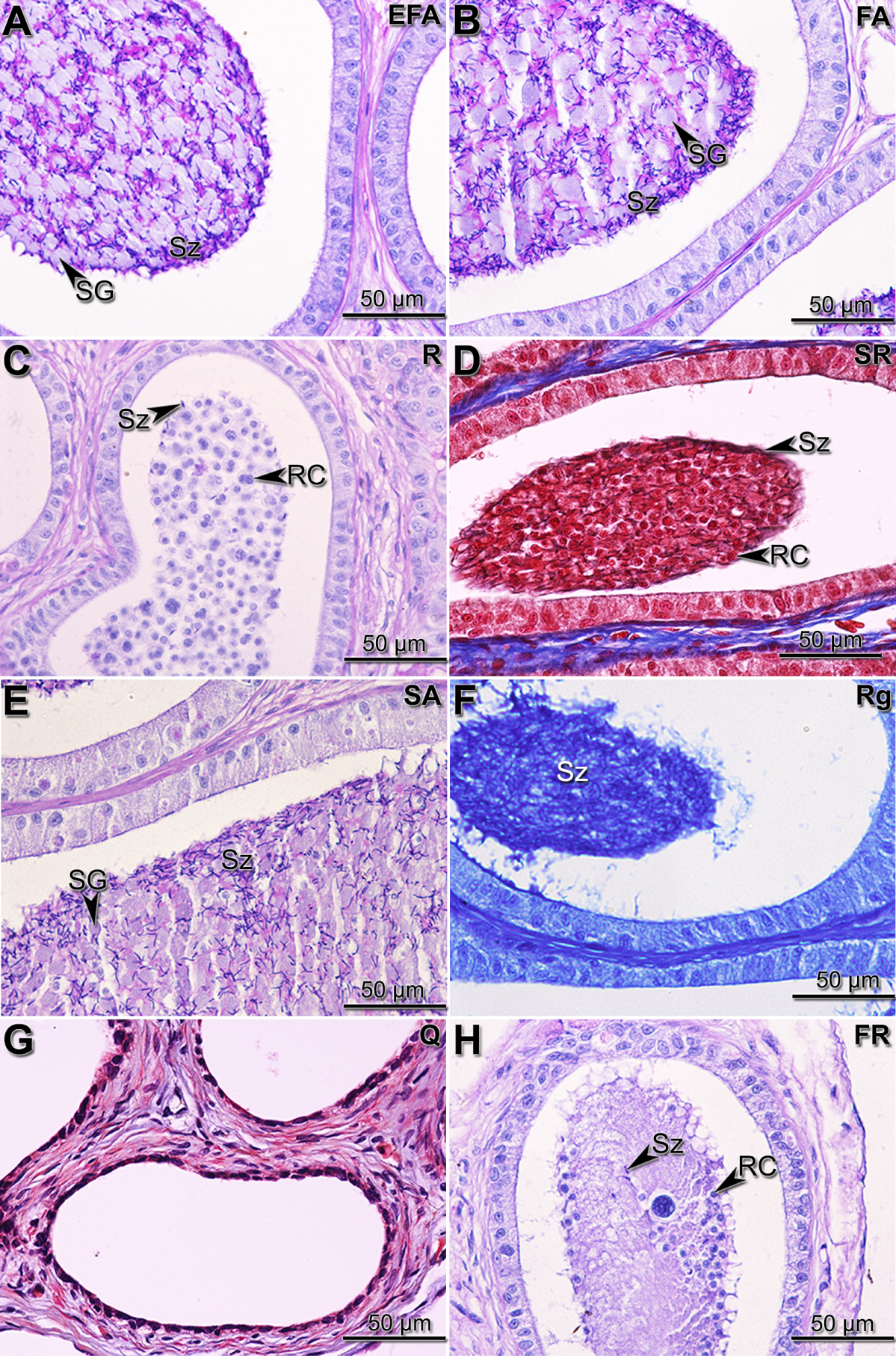


**Fig. S8**


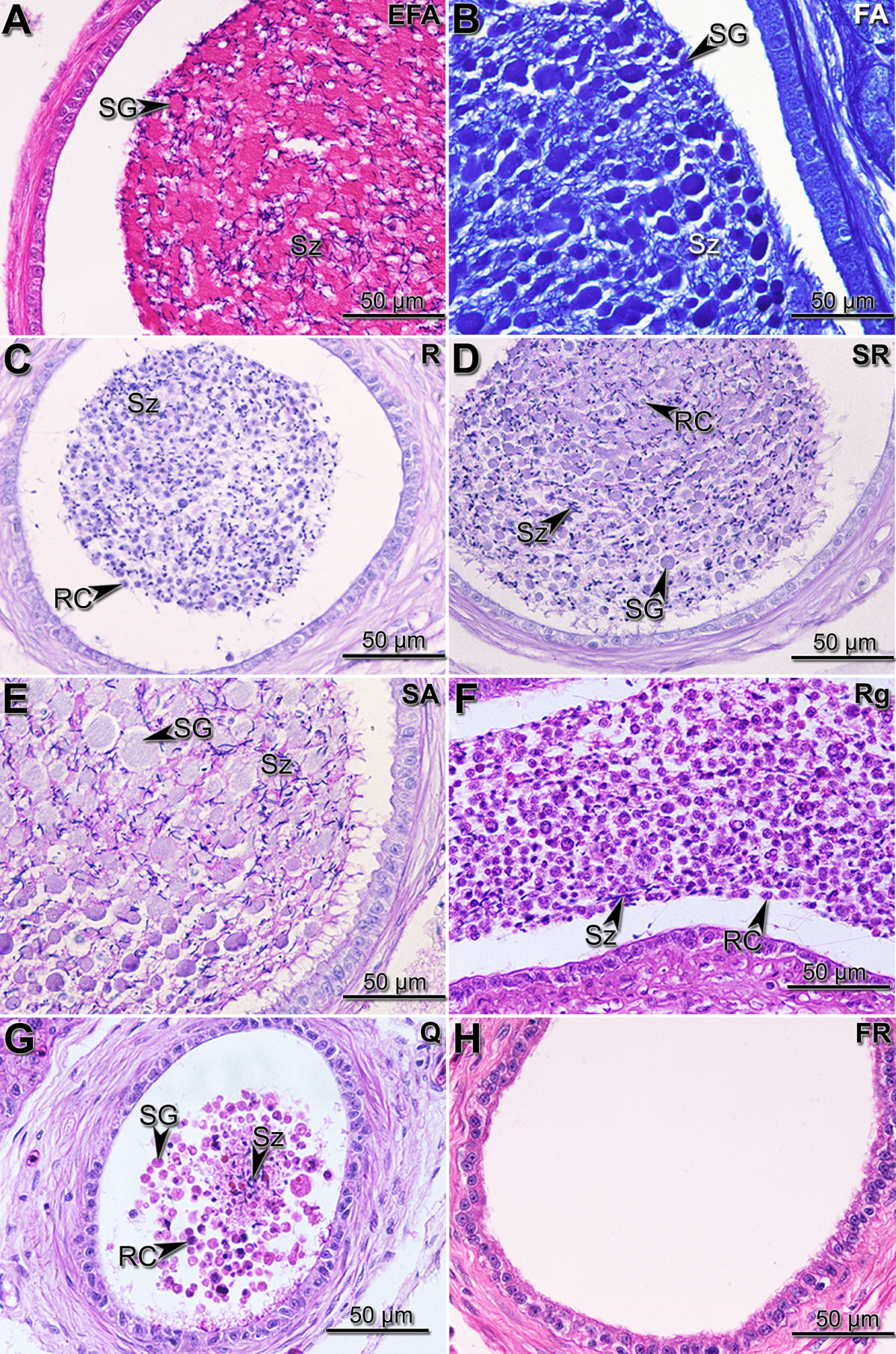


**Fig. S9**


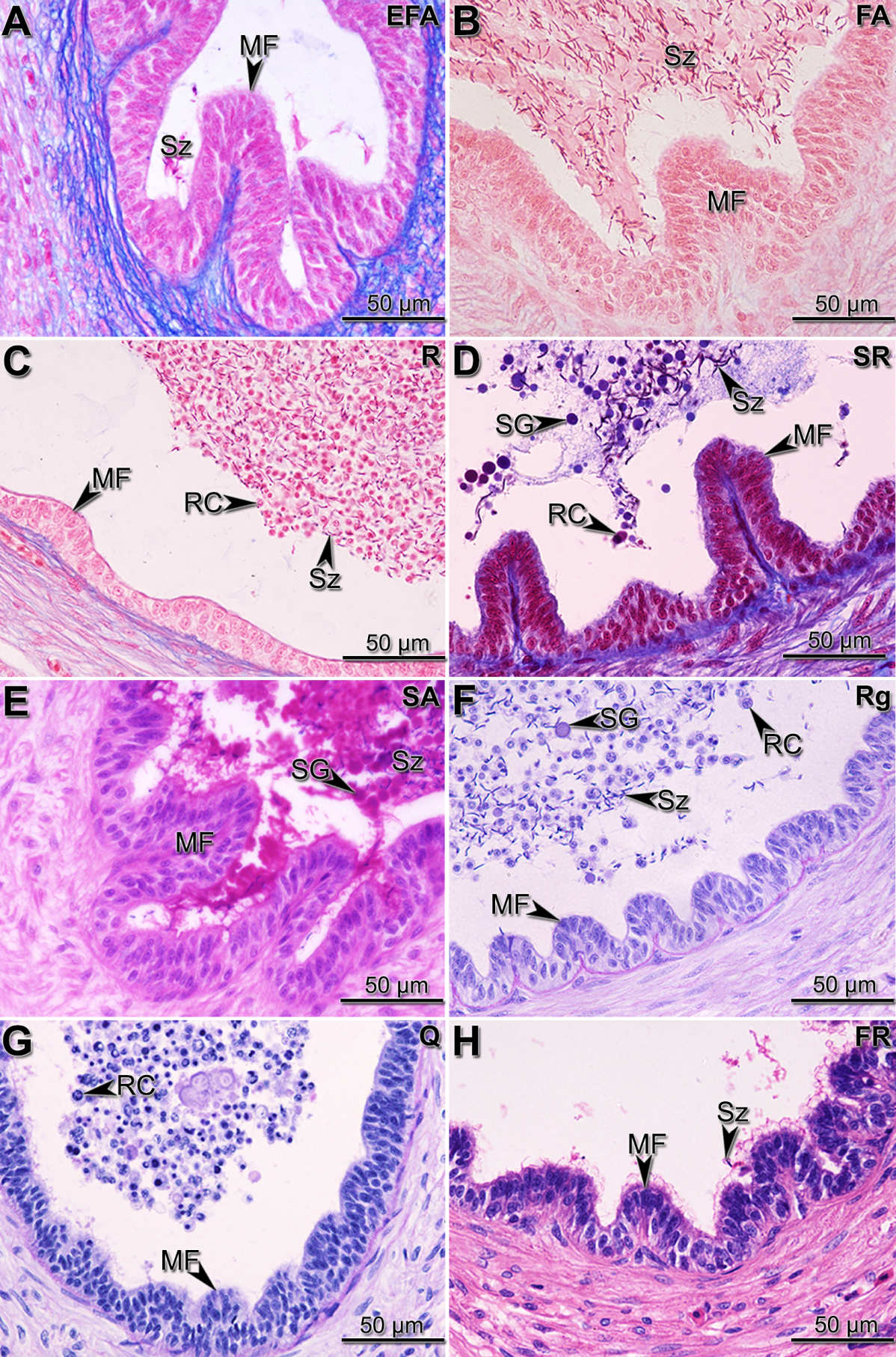


**Fig. S10**
